# The efficacy and safety of olokizumab for rheumatoid arthritis: a systematic review, pairwise, and network meta-analysis

**DOI:** 10.1007/s10067-023-06519-6

**Published:** 2023-02-16

**Authors:** Mohamed Abuelazm, Ahmed Ghanem, Abdelrahman Mahmoud, Aml M. Brakat, Mohamad A. Elzeftawy, Aya Mamdouh Fayoud, Ahmed K. Awad, Basel Abdelazeem

**Affiliations:** 1 Faculty of Medicine, Tanta University, Tanta, Egypt; 2 Cardiology Department, The Lundquist Institute, Torrance, CA USA; 3 Faculty of Medicine, Minia University, Minia, Egypt; 4 Faculty of Medicine, Zagazig University, Zagazig, Egypt; 5 Faculty of Pharmacy, Kafr El-Shiekh University, Kafr El-Shiekh, Egypt; 6 Faculty of Medicine, Ain-Shams University, Cairo, Egypt; 7 Department of Internal Medicine, McLaren Health Care, Flint, MI USA; 8 Department of Internal Medicine, Michigan State University, East Lansing, MI USA

**Keywords:** DMARDs, Meta-analysis, Olokizumab, OKZ, Rheumatoid arthritis, Systematic review

## Abstract

Olokizumab (OKZ) is a novel IL-6 inhibitor that directly targets IL-6 rather than its receptor. We aim to evaluate the efficacy and safety of OKZ for patients with rheumatoid arthritis (RA) and to investigate the optimal treatment regimen. A systematic review, pairwise, and network meta-analysis synthesizing randomized controlled trials (RCTs) from WOS, CENTRAL, SCOPUS, EMBASE, and PubMed until August 31, 2022. We used the risk ratio (RR) and mean difference (MD) for dichotomous and continuous outcomes, respectively, presented with the corresponding 95% confidence interval (CI). We registered our protocol in PROSPERO with ID: CRD42022358082. Five RCTs with 2277 patients were included. OKZ significantly improved the American College of Rheumatology criteria (ACR) 20 (RR: 1.97 with 95% CI [1.49, 2.58], *P* = 0.00001), ACR50 (RR: 3.83 with 95% CI [2.13, 6.87], *P* = 0.00001), ACR70 (RR: 3.83 with 95% CI [2.13, 6.87], *P* = 0.00001), disease activity score 28 based on C-reactive protein (DAS28-CRP) (RR: 3.91 with 95% CI [2.65, 5.79], *P* = 0.00001), clinical disease activity index (CDAI) (RR: 2.80 with 95% CI [1.43, 5.48], *P* = 0.003), and health assessment questionnaire disability index (HAQ-DI) (MD: − 0.28 with 95% CI [− 0.38, − 0.18], *P* = 0.00001) after 12 weeks, compared to placebo. However, OKZ was also associated with a higher incidence of any adverse events (AEs) (RR: 1.15 with 95% CI [1.06, 1.25], P = 0.0005) and AEs leading to drug discontinuation (RR: 1.86 with 95% CI [1.05, 3.29], P = 0.03). OKZ is effective and with acceptable safety profile when administrated with methotrexate in patients with RA not adequately controlled by tumor necrosis factor inhibitors; however, more large-scale RCTs are still required to investigate the optimal dosing, long-term effects, and comparative efficacy versus established biological DMARDs.

**Table:** 

**Key Points** *• OKZ is effective especially with methotrexate in RA patients.*

**Supplementary Information:**

The online version contains supplementary material available at 10.1007/s10067-023-06519-6.

## Introduction

Rheumatoid arthritis (RA) is a multifactorial chronic autoimmune disease that is associated with chronic systemic inflammation, leading to irreversible joint damage and multiple extra-articular morbidities [1, 2]. RA affects females two to three times more than males and can present at any age, with a peak prevalence in the seventh decade of life [3]. Early diagnosis and proper treatment are critical to control RA activity and avoiding permanent disabilities and joint destruction [1].

No curative therapy for RA exists; however, multiple therapeutic options are available to achieve the principle of “treat to target” that was established by the American College of Rheumatology (ACR) [4], which is achieved by either remission or low disease activity and sustaining that for at least 6 months. Different tools are used to evaluate the disease severity. Disease activity score 28 based on C-reactive protein (CRP) and erythrocyte sedimentation rate (ESR) (DAS28-CRP and DAS28-ESR) [5] and ACR 20, 50, and 70 criteria [6] are common indices evaluating RA activity based on symptoms, signs, and laboratory data. Other indices are being used as well, including Health Assessment Questionnaire Disability Index (HAQ-DI) [5] and Clinical Disease Activity Index (CDAI) [7]. The European Alliance of Associations for Rheumatology (EULAR) [8] and ACR [9] recommended disease-modifying anti-rheumatic drugs (DMARDs) and symptomatic treatment as soon as the accurate diagnosis is made to stop the ongoing joint erosions and relieve the patient’s symptoms.

Despite the advancement in the DMARDs, less than 50% of patients with RA are in remission, and 10–20% are refractory to the available treatment options [10]. New treatments targeting different pathways in the disease’s pathophysiology are emerging to cover this unmet gap, including IL-6 inhibitors. IL-6 is a pro-inflammatory cytokine that plays different roles in the pathogenesis of RA. It activates the T-cells, induces B-cell proliferation, induces osteoclast differentiation, enhances angiogenesis, and induces acute phase reactants [2]. The unregulated production of IL-6 in RA is associated with autoimmunity, chronic inflammation, joint edema, and joint destruction [11]. Accordingly, multiple drugs targeting IL-6 started to emerge, including tocilizumab (TCZ), sarilumab, sirukumab, clazakizuma, and olokizumab (OKZ).

Furthermore, IL-6 inhibitors are not used as a monotherapy for RA, but they are combined with conventional synthetic DMARDs (csDMARDs), such as methotrexate (MTX), for a better outcome. When TCZ and MTX were given to patients with inadequate response to TNF-α inhibitors, they achieved rapid and sustained response [12]. Also, sarilumab plus MTX showed significant suppression in the biomarkers of bone resorption and joint damage, compared to placebo plus MTX [13]. Finally, OKZ is a novel IL6 inhibitor with promising treatment outcomes.

To clarify OKZ is a new humanized monoclonal antibody targeting the IL-6 cytokine. Unlike TCZ, it does not target the IL-6 receptor. Instead, it targets specific sites on the IL-6 itself and blocks the formation of the extracellular signaling complex, consequently inhibiting the transmembrane signaling [14]. Two phase II randomized controlled trials (RCTs) [15, 16] reported significant improvement in DAS28-CRP, ACR20, and ACR50 indices in OKZ group, compared to the placebo. In another phase III clinical trial, OKZ combined with MTX was superior to MTX monotherapy and non-inferior to adalimumab combined with MTX [17]. Nasonov et al. [18] also reported that the combination of OKZ and MTX was superior to MTX plus placebo in improving the signs, symptoms, and physical function in RA patients. This was further supported by Feist et al. [19]. Therefore, we performed this systematic review and meta-analysis to synthesize evidence from RCTs on the efficacy and safety of OKZ in patients with RA and to investigate the optimal dosing regimen.

## Methodology

### Protocol registration

This systematic review and meta-analysis was thoroughly conducted in accordance with the Preferred Reporting Items for Systematic Reviews, Meta-analysis, and Network Meta-Analyses (PRISMA [20] and PRISMA NMA [21]) and the Cochrane Handbook of Systematic reviews and meta-analysis [22]. Moreover, this review’s protocol was prospectively published and registered in PROSPERO with ID: CRD42022358082. The thorough PRISMA checklist is in Table S1.

### Data sources and search strategy

Electronic databases, including, PubMed, EMBASE, Web of Science, Scopus, and Cochrane CENTAL were systematically searched by two reviewers (B.A. and M.T.) until August 31, 2022. No filters were used. The comprehensive search terms and findings are elaborated in Table S2.

### Eligibility criteria

We included RCTs with the following PICO criteria: population (P): patients with RA irrespective of their current treatment; intervention (I): OKZ irrespective of the dose (C): placebo (O): primary outcome of this study is the ACR20. Secondary endpoints include ACR50, ACR70, DAS28 CRP < 3.2, CDAI score ≤ 2.8, HAQ-DI score change, DAS28 ESR score change, and finally, safety data, including all-cause mortality, any treatment-emergent adverse events (TAEAs), any serious TAEAs, TAEAs leading to drug discontinuation, gastrointestinal disorder, and infection. All outcomes were measured at 12 and 24 weeks.

Single-arm studies, observational studies, conference abstracts, animal studies, and non-randomized trials were excluded.

### Study selection

After excluding duplicates with Covidence online program, four investigators (A.B., A.F., A.M., and M.A.E.) independently assessed the titles and abstracts of the retrieved articles. Then, they checked the full texts of the relevant records for the previously mentioned eligibility criteria. Disagreements were resolved through discussion.

### Data extraction

Guided by a pilot-tested extraction form, four reviewers (A.B., A.F., A.M., and M.A.E.) independently extracted the following: study characteristics (study design, NCT number, country recruitment duration, total participants, OKZ dose, and frequency of administration, main inclusion criteria, current adjuvant medication, primary outcome and follow up duration); baseline data, including (age, sex, number of patients in each group, basal metabolic index (BMI), rheumatoid factor (RF) positive, anti-CCP positive, DAS28-CRP, CDAI score, HAQ-DI score, tender joint count (TJC), swollen joint count (SJC), patient global assessment of disease activity (PtGA), visual analog scale (VAS), physician global assessment of disease (PGA), MTX dose, Duration of prior MTX use, Glucocorticoid use, Prednisone dose or its equivalent, and Prior exposure to TNF inhibitors). Finally, efficacy and safety outcomes include ACR20, ACR50, and ACR70 response, conversion to DAS28 (CRP) < 3.2 and CDAI score ≤2.8, HAQ-DI, DAS28 (ESR) change from baseline, and adverse events. Disagreements were resolved through discussion.

### Risk of bias and quality assessment

We implemented the revised Cochrane collaboration’s tool for assessing the risk of bias in RCTs (ROB 2) [23], and four reviewers (A.B., A.F., A.M., and M.A.) independently evaluated the included RCTs for the risk of selection, performance, reporting, attrition, and overall biases. Disagreements were resolved by discussion. For the quality of evidence appraisal, two reviewers (M.T. and B.A.) used the Grading of Recommendations Assessment, Development, and Evaluation (GRADE) guidelines [24, 25]. Our decision was rationalized and reported for each outcome. Any disagreements were resolved via discussion.

### Statistical analysis

For the pairwise meta-analysis, we used Revman version 5.4 [26] to pool dichotomous outcomes using risk ratio (RR) and continuous outcomes using mean difference (MD) presented with the corresponding 95% confidence interval (CI). We used the fixed-effect model; however, the random-effect model was utilized in case of significant heterogeneity. *I*^2^ and chi-squared test were used to evaluate the statistical heterogeneity. *P* value < 0.05 was considered significant for the chi-squared test, and *I*^2^ > 50% indicated substantial heterogeneity, in which case sensitivity analysis was conducted by excluding one study each time to determine the source of heterogeneity.

For network meta-analysis, we performed a network meta-analysis using a frequentist framework [21], pooling dichotomous outcomes using risk ratio (RR), and continuous data using mean difference (MD) presented with the corresponding 95% confidence interval (CI). Analysis was performed using the R-software netmeta and netrank package (R version 4.2.0), and meta-insight software [27–29] with statistical inconsistency in between network arms was evaluated by calculating *I*^2^. Finally, we did not investigate the publication bias by funnel plots as we included less than ten RCTs [30].

## Results

### Search results and study selection

**Fig. 1:**
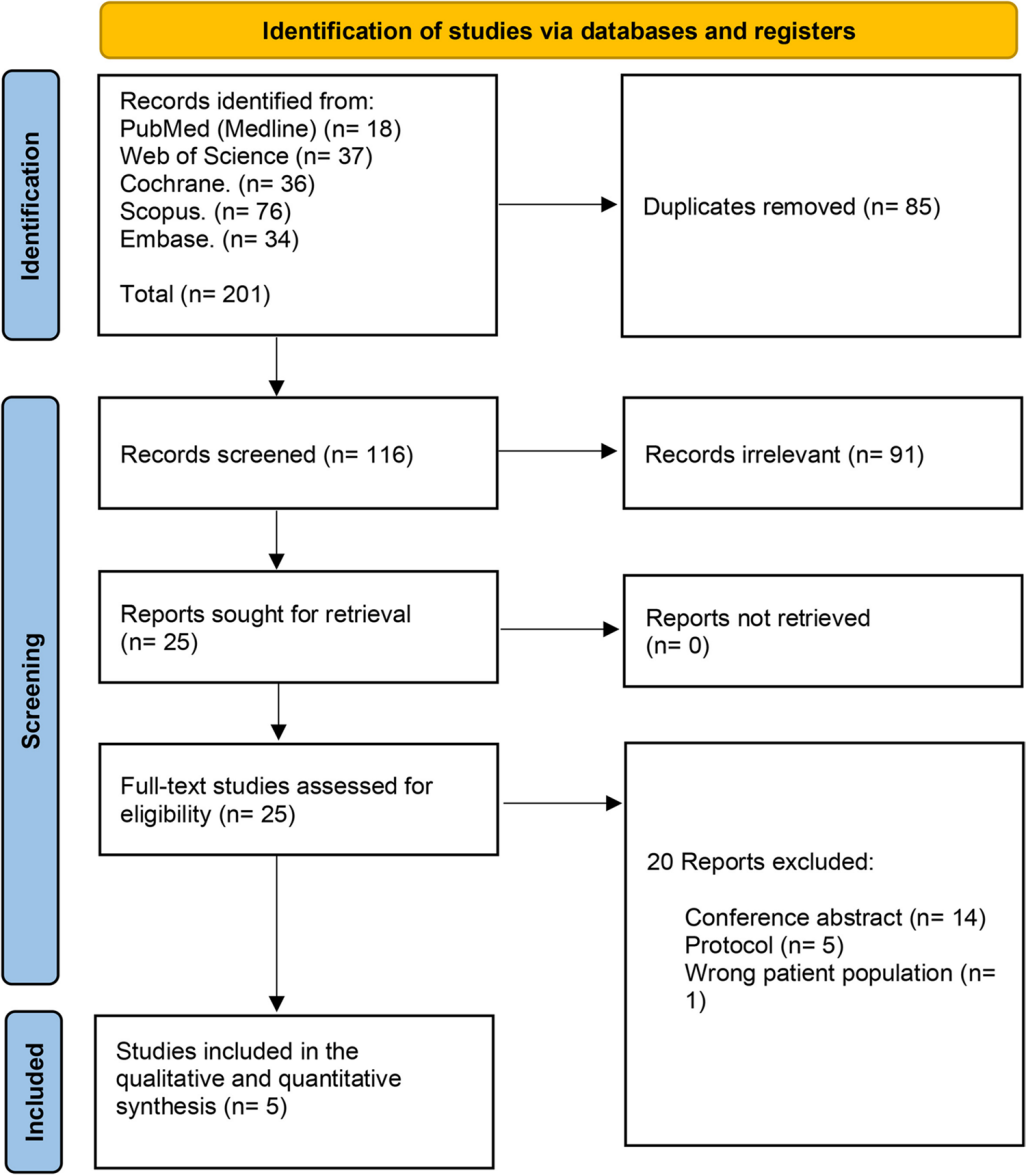
PRISMA flow chart of the screening process

We retrieved 201 records, and then, 85 duplicates were excluded using Covidence, leaving 116 records to be screened. After title and abstract screening, we excluded 91 irrelevant records and proceeded with 25 articles for full-text screening. Finally, we included five RCTs [15–19] (Fig. 1).

### Characteristics of included studies

Five RCTs [15–19] were included with 2277 patients of whom 1749 patients in the OKZ group and 528 patients in the placebo group. Two RCTs were phase II following up patients for 12 weeks [15, 16], while three RCTs were phase III following up patients for 24 weeks [17–19]. Phase III RCTs [17–19] investigated OKZ in the dose of 64 mg given every 2 or 4 weeks, while phase II RCTs [15, 16] were dose-ranging studies investigating the doses of 60, 120, and 240 mg given every 2 or 4 weeks. Summary RCT characteristics and baseline characteristics of the participants are outlined in Tables 1 and 2, respectively.

**Table 1:** Summary characteristics of the included RCTs

Study ID	Study design	Country	*N* of participants		OKZ dose	Adjuvant allowed DMARDs	Main inclusion criteria	Primary outcome
			OKZ	PBO				
Feist et al. 2022 [19]	Multicenter, double-blind, phase III RCT	Argentina, Brazil, Colombia, Czech Republic, Germany, Hungary, South Korea, Mexico, Poland, Russia, and the USA	299	69	64 mg	MTX, steroids not exceeding 10 mg/day prednisone equivalent, and stable dose of NSAIDs	Adult patient with active RA inadequately controlled by TNF inhibitor therapy	ACR20 response at week 12
Genovese et al. 2014 [15]	Multicenter, double-blind, phase IIb, dose-ranging RCT	N/A	132	44	60, 120, and 240 mg	Stable dose of MTX, continued current steroids and NSAIDs	Adult patient with active RA inadequately controlled by MTX and TNF inhibitor therapy	DAS28-CRP change from baseline at week 12
Nasonov et al. 2021 [18]	Multicenter, double-blind, phase III RCT	Russia, Belarus, and Bulgaria	285	143	64 mg	MTX, steroids not exceeding 10 mg/day prednisone equivalent, and stable dose of NSAIDs	Adult patient with active RA inadequately controlled by MTX	ACR20 response at week 12
Smolen et al. 2022 [17]	Multicenter, double-blind, phase III RCT	Argentina, Bulgaria, Brazil, Colombia, Czech Republic, Germany, Estonia, the UK, Hungary, South Korea, Lithuania, Latvia, Mexico, Poland, Romania, Russia, Taiwan, and the USA	943	243	64 mg	MTX, steroids not exceeding 10 mg/day prednisone equivalent, and stable dose of NSAIDs	Adult patient with active RA inadequately controlled by MTX	ACR20 response at week 12
Takeuchi et al. 2016 [16]	Multicenter, double-blind, phase II RCT	Japan, Korea, and Taiwan	90	29	60, 120, and 240 mg	MTX, steroids not exceeding 10 mg/day prednisone equivalent, and stable dose of NSAIDs	Adult patient with active RA inadequately controlled by MTX and TNF inhibitor therapy	DAS28-CRP change from baseline at week 12

*RCT*, randomized controlled trial; *OKZ*, olokizumab; *PBO*, placebo; *N*, number; *MTX*, methotrexate; *TNF*, tumor necrosis factor; *NSAIDs*, non-steroidal anti-inflammatory drugs; *DMARDs*, disease-modifying anti-rheumatic drugs; *ACR*, American College of Rheumatology; *DAS28-CRP*, disease activity score 28 based on C-reactive protein; *mg*, milligram; *N/A*, not available

**Table 2:** Baseline characteristics of the participants

Study ID	Age (years) mean (SD)			Gender (female) *N* (%)			BMI, mean (SD)			RF positive, *N* (%)			Anti-CCP positive, *N* (%)		
	OKZ		PBO	OKZ		PBO	OKZ		PBO	OKZ		PBO	OKZ		PBO
	q2w	q4w		q2w	q4w		q2w	q4w		q2w	q4w		q2w	q4w	
Feist et al. 2022 [19]	53.4 (12.7)	53.9 (11.7)	53.0 (13.7)	122 (88.4)	130 (80.7)	55 (79.7)	28.8 (7.0);	29.2 (6.0)	28.4 (5.6)	105 (76.1)	128 (79.5)	55 (79.7)	96 (69.6)	124 (77.0)	58 (84.1)
Genovese et al. 2014 [15]	60 mg: 55.5; 120 mg: 53.1; 240 mg: 55.5	60 mg: 52.64; 120 mg: 53.52; 240 mg: 54.55	Q2W: 59.63; Q4W: 58.18	60 mg: 16 (80); 120 mg: 19 (86.4); 240 mg: 21 (91.3)	60 mg: 20 (90.9); 120 mg: 20 (87); 240 mg: 17 (77.3)	Q2W: 19 (86.4) Q4W: 17 (77.3)	N/A	N/A	N/A	N/A	N/A	N/A	N/A	N/A	N/A
Nasonov et al. 2021 [18]	52.0 (11.8)	49.1 (12.1)	52.7 (11.3)	116 (81.1)	118 (83.1)	120 (83.9)	26.6 (5.1)	26.4 (5.5)	26.9 (5.0)	115 (80.4)	122 (85.9)	127 (88.8)	110 (76.9)	115 (81.0)	117 (81.8)
Smolen et al. 2022 [17]	53.3 (11.9)	53.7 (12.1)	54.7 (11.9)	352 (75.9)	378 (78.9)	190 (78.2)	28.7 (6.1)	28.7 (6.3)	28.6 (6.6)	352 (75.9)	355 (74.1)	181 (74.5)	355 (76.5)	361 (75.4)	188 (77.4)
Takeuchi et al. 2016 [16]	N/A	60 mg: 53.9 (10.6); 120 mg: 55.7 (10.8); 240 mg: 56.7 (11.0)	52.6 (11.3)	N/A	60 mg: 31 (96.6); 120 mg: 26 (81.3); 240 mg: 21 (80.8)	25 (86.2)	N/A	N/A	N/A	N/A	N/A	N/A	N/A	N/A	N/A

Study ID	DAS28-CRP, mean (SD)			CDAI score, mean (SD)			HAQ-DI score, mean (SD)			TJC, mean (SD)			SJC, mean (SD)		
	OKZ		PBO	OKZ		PBO	OKZ		PBO	OKZ		PBO	OKZ		PBO
	q2w	q4w		q2w	q4w		q2w	q4w		q2w	q4w		q2w	q4w	
Feist et al. 2022 [19]	5.9 (0.9)	6.0 (0.8)	6.2 (0.9)	40.7 (12.5)	41.7 (10.6)	44.4 (11.7)	1.8 (0.6)	1.8 (0.6)	1.8 (0.6)	26.0 (13.7)	25.6 (12.8)	28.2 (13.7)	16.8 (8.2)	17.0 (7.8)	19.3 (9.5)
Genovese et al. 2014 [15]	60 mg: 5.57; 120 mg: 5.96; 240 mg: 5.94	60 mg: 6.14; 120 mg: 5.61; 240 mg: 5.83	q2W: 5.53; Q4W: 5.69	60 mg: 40.27 (11);120 mg: 44.5 (12.9); 240 mg: 43.2 (11.55)	60 mg: 46 (12); 120 mg: 38.5 (11.35); 240 mg: 41.1 (8.75)	Q2W: 37.1 (8.3); Q4W: 37.9 (10.25)	60 mg: 1.5 (0.4); 120 mg: 1.62 (0.6); 240 mg: 1.8 (0.5)	60 mg: 1.7 (0.7); 120 mg: 1.55 (0.65); 240 mg: 1.55 (0.7)	Q2W: 1.5 (0.58); Q4W: 1.29 (0.6)	60 mg: 28.5 (14.75); 120 mg: 30.75 (13.5); 240 mg: 30.75 (11.25)	60 mg: 37.25 (14.75); 120 mg: 30.65 (12.5); 240 mg: 30.36 (12.2)	Q2W: 32.9 (11.85); Q4W: 27.5 (12.5)	60 mg: 22.25 (11); 120 mg: 23.25 (13); 240 mg: 21.85 (8.65)	60 mg: 26.75 (13); 120 mg: 17 (7.8); 240 mg: 18.5 (8.23)	Q2W: 15 (6); Q4W: 18.76 (10.18)
Nasonov et al. 2021 [18]	6.0 (0.7)	5.9 (0.7)	6.0 (0.8)	40.5 (9.8)	38.7 (9.4)	40.4 (10.5)	1.74 (0.47)	1.64 (0.50)	1.78 (0.49)	24.4 (11.4)	22.2 (10.3)	24.0 (11.3)	14.8 (6.5) O	14.5 (6.7)	14.6 (6.9)
Smolen et al. 2022 [17]	5.9 (0.8)	5.8 (0.8)	5.8 (0.8)	39.4 (11.0)	39.4 (11.3)	38.7 (11.4)	1.73 (0.58)	1.69 (0.60)	1.71 (0.62)	23.9 (12.5)	23.6 (12.9)	22.4 (12.3)	14.6 (7.3)	15.4 (8.8)	14.9 (8.5)
Takeuchi et al. 2016 [16]	N/A	60 mg: 5.5 (0.925); 120 mg: 5.2 (0.875);240 mg: 5.3 (0.75)	5.4 (1.05)	N/A	60 mg: 39.35 (15.3); 120 mg: 27.3 (8.65); 240 mg: 29.8 (6.925)	35.6 (11.45)	N/A	60 mg: 1.19 (0.7); 120 mg: 1.25 (0.75); 240 mg: 0.88 (0.575)	1.13 (0.6)	N/A	60 mg: 14.5 (15.5); 120 mg: 12.5 (11.75); 240 mg: 13 (7.25)	16 (9.25)	N/A	60 mg: 12.5 (9); 120 mg: 10 (5.25); 240 mg: 12 (6.25)	12 (9.25)

*OKZ*, olokizumab; *PBO*, placebo; *q2w*, every 2 weeks; *q4w*, every 4 weeks; *BMI*, basal metabolic index; *RF*, rheumatoid factor; *CCP*. cyclic citrullinated peptide; *DAS28-CRP*, disease activity score 28 based on C-reactive protein; *HAQ-DI*, health assessment questionnaire disability index; *CDAI*, clinical disease activity index score; *SJC*, swollen joint count; *TJ*, tender joint count; *N*, number; *SD*, standard deviation; *N/A*, not available

### Risk of bias and quality of evidence

**Fig. 2:**
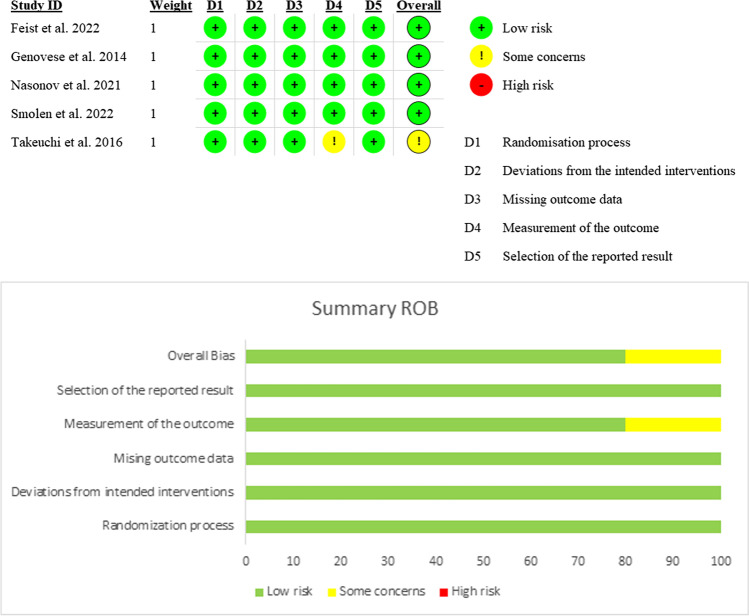
Quality assessment of risk of bias in the included trials. The upper panel presents a schematic representation of risks (low = red, unclear = yellow, and high = red) for specific types of biases of each of the studies in the review. The lower panel presents risks (low = red, unclear = yellow, and high = red) for the subtypes of biases of the combination of studies included in this review

All of the included RCTs [15, 17–19] showed a low risk of overall bias, except Takeuchi et al. [16], with some concerns due to not available information about outcome assessor blinding (Fig. 2). The quality of evidence is outlined in a GRADE evidence profile (Table S3).

### Efficacy outcomes

#### ACR20 response

**Fig. 3:**
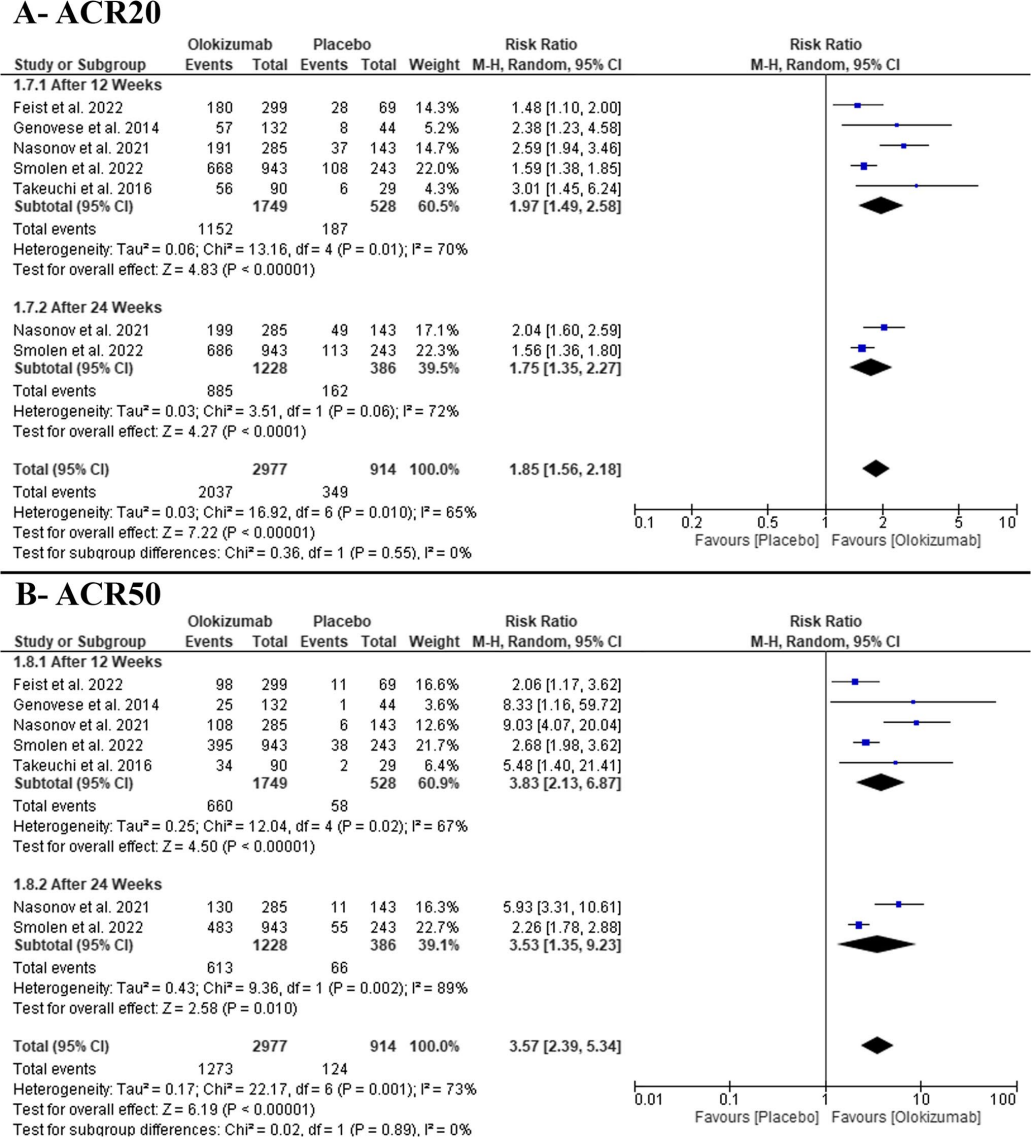
Forest plot of the efficacy outcomes (**A** ACR20, **B** ACR50); RR, risk ratio; CI, confidence interval

In the pairwise meta-analysis, the pooled RR favored OKZ over placebo after 12 weeks (RR: 1.97 with 95% CI [1.49, 2.58], *P* = 0.00001) (moderate-quality evidence) and after 24 weeks (RR: 1.75 with 95% CI [1.35, 2.27], *P* = 0.0001) (moderate-quality evidence) (Fig. 3A, Table S3). The pooled studies were heterogenous (*P* = 0.01, *I*^2^ = 70%) and (*P* = 0.06, *I*^2^ = 72%), respectively. Heterogeneity after 12 weeks was best resolved after excluding Nasonov et al. (2021) (*P* = 0.21, *I*^2^ = 34%) (Table S4).

In the network meta-analysis, all OKZ regimens were significantly associated with greater ACR20 response, compared to placebo either after 12 or 24 weeks, with low heterogeneity observed of *I*^2^ = 37% and *I*^2^ = 43%, respectively (Table 3, Figs. S1-6).

**Table 3:** Ranking table for all our network meta-analyses’ outcomes

ACR20 response after 12 weeks					
Placebo					
0.57 [0.47; 0.70]	OKZ 64 Q2W				
0.55 [0.45; 0.68]	0.97 [0.82; 1.14]	OKZ 64 Q4W			
0.39 [0.23; 0.68]	0.68 [0.38; 1.22]	0.71 [0.39; 1.26]	OKZ 120 Q4W	0.96 [0.66; 1.40]	0.91 [0.65; 1.29]
0.37 [0.21; 0.66]	0.65 [0.36; 1.18]	0.68 [0.37; 1.22]	0.96 [0.66; 1.39]	OKZ 60 Q4W	0.94 [0.65; 1.36]
0.35 [0.20; 0.61]	0.62 [0.35; 1.10]	0.64 [0.36; 1.14]	0.90 [0.64; 1.28]	0.94 [0.66; 1.36]	OKZ 240 Q4W
ACR20 response after 24 weeks					
Placebo					
0.59 [0.51; 0.69]	OKZ 64 Q4W	0.99 [0.90; 1.09]			
0.59 [0.51; 0.68]	0.99 [0.90; 1.09]	OKZ 64 Q2W			
ACR50 response after 12 weeks					
Placebo					
0.34 [0.25; 0.47]	OKZ 64 Q2W				
0.34 [0.24; 0.46]	0.99 [0.80; 1.22]	OKZ 64 Q4W			
0.19 [0.06; 0.62]	0.55 [0.16; 1.88]	0.56 [0.16; 1.91]	OKZ 60 Q4W	0.89 [0.50; 1.59]	0.85 [0.47; 1.54]
0.17 [0.05; 0.54]	0.49 [0.14; 1.65]	0.49 [0.15; 1.67]	0.88 [0.49; 1.59]	OKZ 120 Q4W	0.95 [0.54; 1.69]
0.16 [0.05; 0.52]	0.46 [0.14; 1.58]	0.47 [0.14; 1.60]	0.84 [0.47; 1.52]	0.95 [0.54; 1.69]	OKZ 240 Q4W
ACR50 response after 24 weeks					
Placebo					
0.35 [0.23; 0.52]	OKZ 64 Q2W				
0.32 [0.21; 0.48]	0.92 [0.69; 1.23]	OKZ 64 Q4W			
ACR70 response after 12 weeks					
Placebo					
0.37 [0.04; 3.56]	OKZ 60 Q4W				
0.28 [0.10; 0.78]	0.77 [0.06; 9.25]	OKZ 64 Q4W			
0.22 [0.03; 2.01]	0.61 [0.14; 2.73]	0.79 [0.07; 8.76]	OKZ 240 Q4W		
0.19 [0.07; 0.52]	0.53 [0.04; 6.27]	0.68 [0.40; 1.17]	0.87 [0.08; 9.58]	OKZ 64 Q2W	
0.16 [0.02; 1.29]	0.43 [0.11; 1.68]	0.55 [0.05; 5.68]	0.70 [0.20; 2.41]	0.81 [0.08; 8.30]	OKZ 120 Q4W
ACR70 response after 24 weeks					
Placebo					
0.32 [0.19; 0.54]	OKZ 64 Q4W				
0.31 [0.19; 0.52]	0.96 [0.69; 1.34]	OKZ 64 Q2W			
DAS28-CRP ≤ 3.2 after 12 weeks					
Placebo					
0.37 [0.17; 0.82]	OKZ 60 Q4W				
0.32 [0.15; 0.68]	0.87 [0.51; 1.48]	OKZ 120 Q4W			
0.26 [0.18; 0.38]	0.70 [0.29; 1.69]	0.81 [0.35; 1.89]	OKZ 64 Q4W		
0.26 [0.12; 0.54]	0.70 [0.42; 1.16]	0.81 [0.51; 1.28]	1.00 [0.43; 2.31]	OKZ 240 Q4W	
0.24 [0.17; 0.36]	0.66 [0.27; 1.61]	0.77 [0.33; 1.80]	0.95 [0.74; 1.23]	0.95 [0.41; 2.20]	OKZ 64 Q2W
DAS28-CRP ≤ 3.2 after 24 weeks					
Placebo					
0.28 [0.21; 0.37]	OKZ 64 Q4W				
0.23 [0.19; 0.30]	0.85 [0.70; 1.03]	OKZ 64 Q2W			
CDAI score of ≤ 2.8 after 12 weeks					
Placebo					
0.40 [0.20; 0.81]	OKZ 64 Q4W				
0.35 [0.17; 0.70]	0.86 [0.58; 1.27]	OKZ 64 Q2W			
CDAI score of ≤ 2.8 after 24 weeks					
Placebo					
0.32 [0.15; 0.70]	OKZ 64 Q4W				
0.29 [0.13; 0.63]	0.89 [0.57; 1.41]	OKZ 64 Q2W			
HAQ-DI					
OKZ 60 Q4W					
− 0.39 [− 0.64; − 0.15]	OKZ 120 Q4W				
− 0.53 [− 0.82; − 0.24]	− 0.13 [− 0.42; 0.15]	OKZ 64 Q2W			
− 0.56 [− 0.85; − 0.28]	− 0.17 [− 0.45; 0.11]	− 0.04 [− 0.12; 0.05]	OKZ 64 Q4W		
− 0.61 [− 0.86; − 0.37]	− 0.22 [− 0.46; 0.02]	− 0.09 [− 0.37; 0.20]	− 0.05 [− 0.33; 0.23]	OKZ 240 Q4W	
− 0.78 [− 1.05; − 0.50]	− 0.38 [− 0.65; − 0.11]	− 0.25 [− 0.34; − 0.16]	− 0.21 [− 0.30; − 0.12]	− 0.16 [− 0.43; 0.10]	Placebo
Any TEAEs					
Placebo					
0.96 [0.79; 1.17]	OKZ 240 Q4W				
0.93 [0.77; 1.12]	0.96 [0.81; 1.15]	OKZ 120 Q4W			
0.89 [0.74; 1.07]	0.92 [0.77; 1.11]	0.96 [0.81; 1.14]	OKZ 60 Q4W		
0.88 [0.80; 0.97]	0.92 [0.74; 1.14]	0.95 [0.77; 1.17]	0.99 [0.81; 1.22]	OKZ 64 Q2W	
0.87 [0.79; 0.96]	0.91 [0.73; 1.13]	0.94 [0.77; 1.16]	0.98 [0.80; 1.21]	0.99 [0.92; 1.07]	OKZ 64 Q4W
Any TEAEs leading to drug discontinuation					
OKZ 120 Q4W	0.45 [0.04; 4.74]	0.50 [0.05; 5.24]	0.41 [0.04; 4.23]		
0.45 [0.04; 4.74]	Placebo	1.10 [0.17; 7.34]	0.90 [0.14; 5.92]	0.61 [0.31; 1.20]	0.48 [0.25; 0.93]
0.50 [0.05; 5.24]	1.10 [0.17; 7.34]	OKZ 60 Q4W	0.81 [0.12; 5.38]		
0.41 [0.04; 4.23]	0.90 [0.14; 5.92]	0.81 [0.12; 5.38]	OKZ 240 Q4W		
0.27 [0.02; 3.11]	0.60 [0.31; 1.17]	0.54 [0.07; 4.05]	0.67 [0.09; 4.94]	OKZ 64 Q2W	0.82 [0.53; 1.26]
0.22 [0.02; 2.55]	0.49 [0.26; 0.95]	0.45 [0.06; 3.31]	0.55 [0.07; 4.05]	0.82 [0.53; 1.27]	OKZ 64 Q4W
Any TESAEs					
OKZ 60 Q4W					
0.92 [0.19; 4.55]	OKZ 240 Q4W				
0.82 [0.16; 4.30]	0.89 [0.20; 3.88]	OKZ 120 Q4W			
0.61 [0.14; 2.64]	0.66 [0.18; 2.43]	0.74 [0.20; 2.84]	Placebo		
0.59 [0.12; 2.85]	0.64 [0.15; 2.66]	0.72 [0.17; 3.10]	0.97 [0.54; 1.73]	OKZ 64 Q4W	
0.46 [0.10; 2.20]	0.50 [0.12; 2.06]	0.56 [0.13; 2.39]	0.75 [0.42; 1.33]	0.78 [0.49; 1.23]	OKZ 64 Q2W
Any-cause mortality					
OKZ 64 Q4W					
1.02 [0.09; 11.18]	Placebo				
0.65 [0.11; 3.85]	0.64 [0.07; 6.07]	OKZ 64 Q2W			
Gastrointestinal disorders					
Placebo					
1.03 [0.54; 1.95]	OKZ 120 Q4W				
0.93 [0.47; 1.85]	0.90 [0.46; 1.77]	OKZ 60 Q4W			
0.85 [0.55; 1.30]	0.82 [0.38; 1.78]	0.91 [0.40; 2.05]	OKZ 64 Q4W		
0.84 [0.45; 1.54]	0.81 [0.44; 1.51]	0.90 [0.46; 1.77]	0.99 [0.47; 2.09]	OKZ 240 Q4W	
0.76 [0.50; 1.16]	0.74 [0.34; 1.60]	0.82 [0.36; 1.84]	0.90 [0.65; 1.24]	0.91 [0.43; 1.92]	OKZ 64 Q2W
Infections					
OKZ 60 Q4W					
0.93 [0.52; 1.65]	OKZ 64 Q2W				
0.85 [0.50; 1.44]	0.91 [0.55; 1.50]	OKZ 240 Q4W			
0.84 [0.48; 1.50]	0.91 [0.77; 1.07]	1.00 [0.61; 1.64]	OKZ 64 Q4W		
0.79 [0.48; 1.32]	0.85 [0.52; 1.40]	0.94 [0.60; 1.45]	0.94 [0.57; 1.53]	OKZ 120 Q4W	
0.81 [0.47; 1.39]	0.87 [0.72; 1.05]	0.95 [0.60; 1.51]	0.96 [0.79; 1.15]	1.02 [0.65; 1.60]	Placebo

*OKZ*, olokizumab; *TAEAs*, any treatment emergent adverse event; *DAS28-CRP*, disease activity score 28 based on C-reactive protein; *HAQ-DI*, health assessment questionnaire disability index; *CDAI*, clinical disease activity index score; *ACR*, American College of Rheumatology

All data are reported in risk ratio (RR) and 95% confidence interval (CI)

#### ACR50 response

In the pairwise meta-analysis, the pooled RR favored OKZ over placebo after 12 weeks (RR: 3.83 with 95% CI [2.13, 6.87], *P* = 0.00001) (moderate-quality evidence) and after 24 weeks (RR: 3.53 with 95% CI [1.35, 9.23], *P* = 0.01) (low-quality evidence) (Fig. 3B, Table S3). The pooled studies were heterogenous (*P* = 0.02, *I*^2^ = 67%) and (P = 0.002, I^2^ = 89%), respectively. Heterogeneity after 12 weeks was best resolved after excluding Nasonov et al. (2021) (*P* = 0.36, *I*^2^ = 7%) (Table S4).

In the network meta-analysis, all OKZ regimens were significantly associated with greater ACR50 response, compared to placebo either after 12 or 24 weeks, with low heterogeneity observed of *I*^2^ = 22% and *I*^2^ = 34%, respectively (Table 3, Figs. S7-12).

#### ACR70 response

**Fig. 4:**
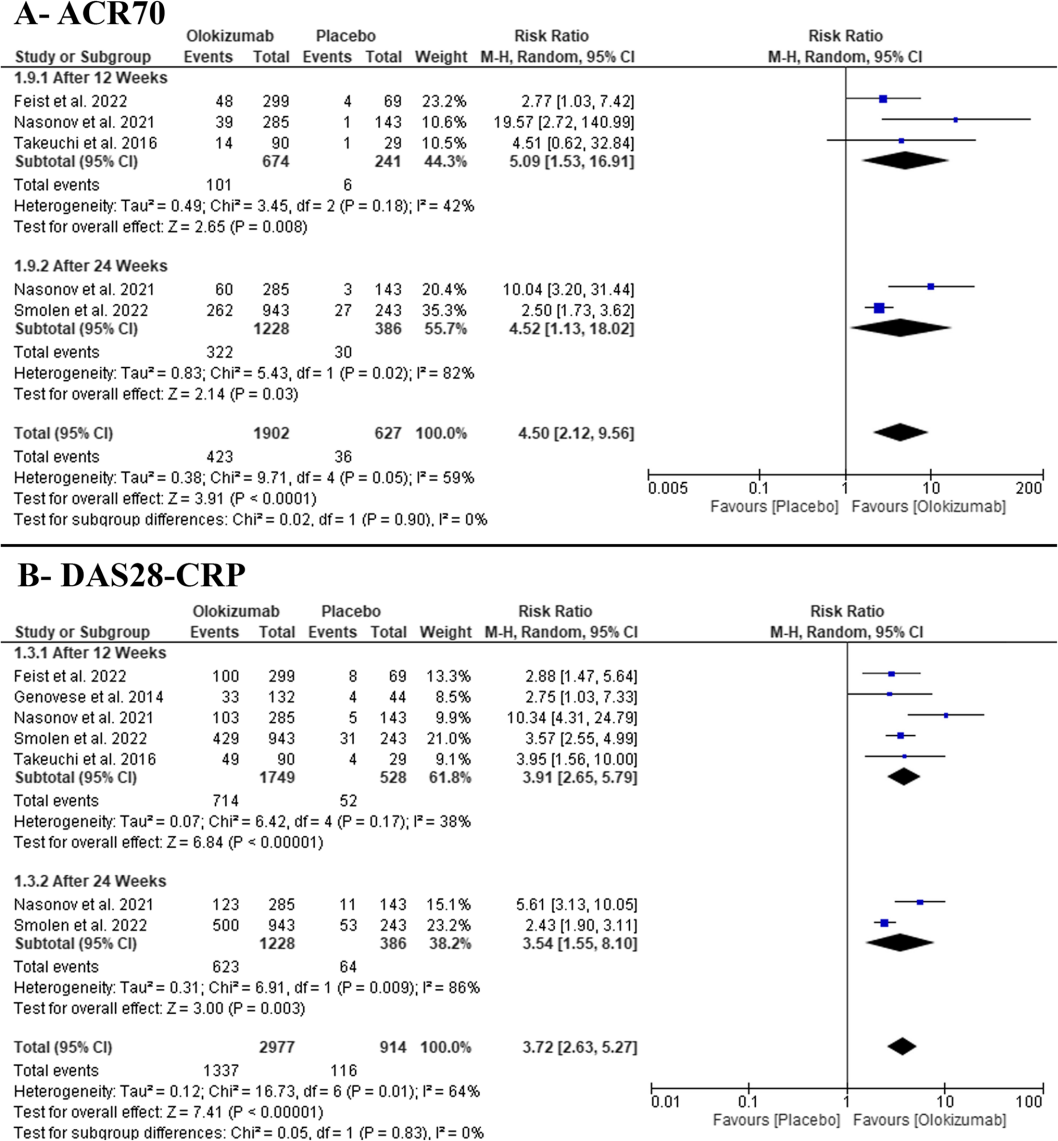
Forest plot of the efficacy outcomes (**A** ACR70, **B** DAS28-CPR); RR, risk ratio; CI, confidence interval

In the pairwise meta-analysis, the pooled RR favored OKZ over placebo after 12 weeks (RR: 3.83 with 95% CI [2.13, 6.87], *P* = 0.00001) (moderate-quality evidence) and after 24 weeks (RR: 5.09 with 95% CI [1.53, 16.91], *P* = 0.008) (low-quality evidence) (Fig. 4A, Table S3). The pooled studies were homogenous after 12 weeks (*P* = 0.18, *I*^2^ = 42%) and heterogenous after 24 weeks (*P* = 0.02, *I*^2^ = 82%).

In the network meta-analysis, OKZ 64 Q2w and Q4w regimens were significantly associated with greater ACR70 response, compared to placebo either after 12 or 24 weeks with, substantial heterogeneity observed of *I*^2^ = 52% and 73%, respectively (Table 3, Figs. S13-18).

#### DAS28-CRP ≤ 3.2

In the pairwise meta-analysis, the pooled RR favored OKZ over placebo after 12 weeks (RR: 3.91 with 95% CI [2.65, 5.79], *P* = 0.00001) (high-quality evidence) and after 24 weeks (RR: 3.54 with 95% CI [1.55, 8.10], *P* = 0.003) (low-quality evidence) (Fig. 4B, Table S3). The pooled studies were homogenous after 12 weeks (*P* = 0.17, *I*^2^ = 38%) and heterogenous after 24 weeks (*P* = 0.009, *I*^2^ = 86%).

In the network meta-analysis, all OKZ regimens were significantly associated with improved DAS28-CRP, compared to placebo either after 12 or 24 weeks, with low heterogeneity observed of *I*^2^ = 43% for 12 weeks, yet substantial heterogeneity of *I*^2^ = 76% for 24 weeks (Table 3, Figs. S19-24).

#### CDAI score of ≤ 2.8

In the pairwise meta-analysis, the pooled RR favored OKZ over placebo after 12 weeks (RR: 2.80 with 95% CI [1.43, 5.48], *P* = 0.003) (moderate-quality evidence) and after 24 weeks (RR: 3.67 with 95% CI [2.01, 6.72], *P* = 0.0001) (low-quality evidence) (Fig. S25, Table S3). The pooled studies were homogenous after 12 weeks (*P* = 0.62, *I*^2^ = 0%) and after 24 weeks (*P* = 0.13, *I*^2^ = 57%).

In the network meta-analysis, all OKZ regimens were significantly associated with improved CDAI score, compared to placebo either after 12 or 24 weeks, with low heterogeneity observed of *I*^2^ = 11% and *I*^2^ = 24%, respectively (Table 3, Figs. S26-31).

#### HAQ-DI score change after 12 weeks

In the pairwise meta-analysis, the pooled MD favored OKZ over placebo (MD: − 0.28 with 95% CI [− 0.38, − 0.18], *P* = 0.00001) (moderate-quality evidence) (Fig. S32, Table S3). The pooled studies were heterogenous (*P* = 0.002, *I*^2^ = 76%). A sensitivity analysis was conducted to determine the source of heterogeneity, but it was not resolved by sensitivity analysis (Table S4).

In the network meta-analysis, all treatment regimens were associated with a reduction in HAQ-DI score change after 12 weeks, except for OKZ 240 Q4W (MD: − 0.16 with 95% CI [− 0.43, 0.10]), with substantial heterogeneity observed of *I*^2^ = 54% (Table 3, Figs. S33-35).

#### DAS28-ESR score change after 12 weeks

We found no difference between OKZ and placebo (MD: − 3.69 with 95% CI [− 8.13, 0.75], *P* = 0.1) (very low-quality evidence) (Fig. S36). The pooled studies were heterogenous (*P* = 0.0001, *I*^2^ = 94%).

### Safety outcomes

#### Any TEAEs

**Fig. 5:**
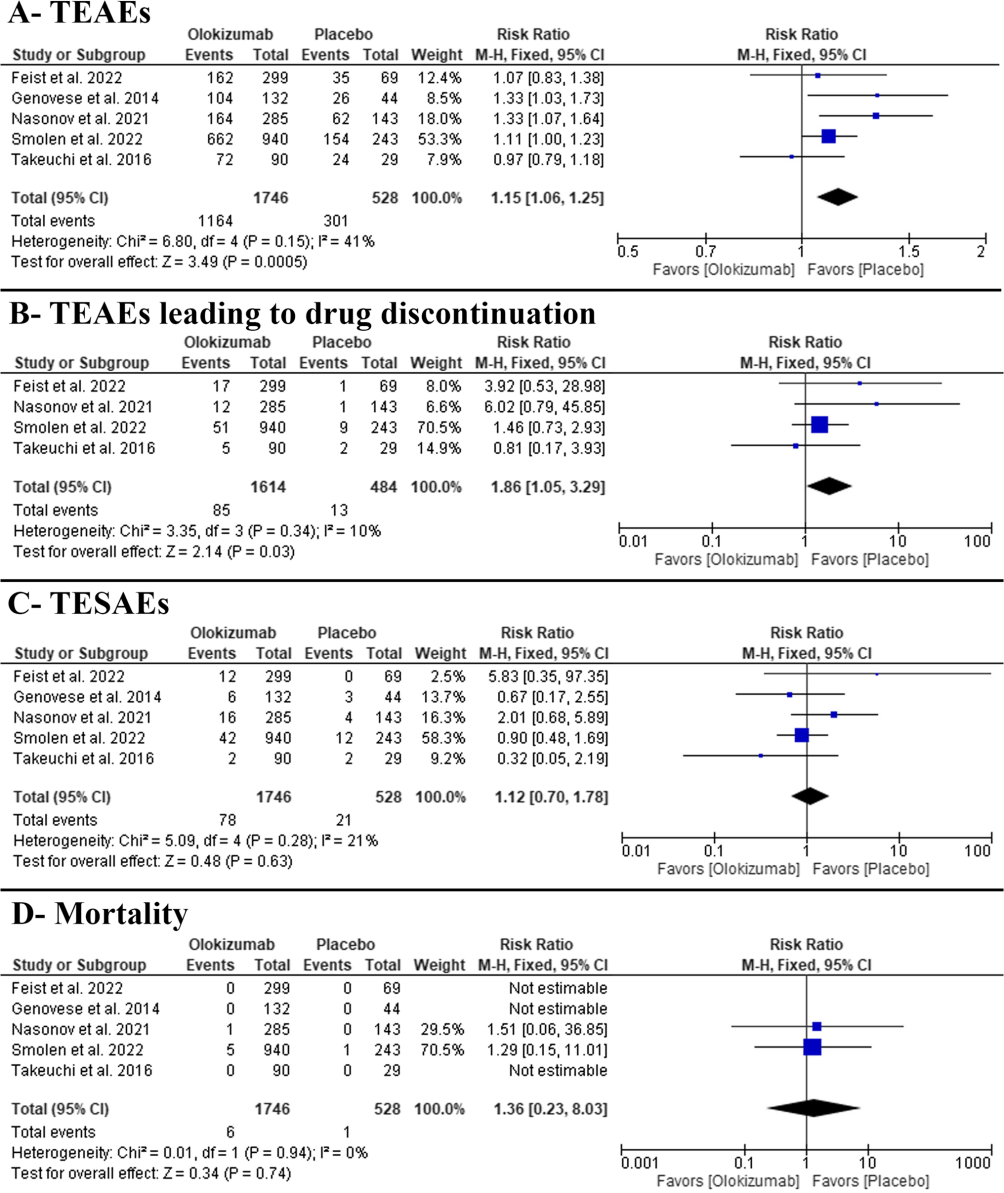
Forest plot of the safety outcomes (**A** TEAEs, **B** TEAEs leading to drug discontinuation, **C** TESAEs, and **D** all-cause mortality); RR, risk ratio; CI, confidence interval

In the pairwise meta-analysis, OKZ was significantly associated with more incidence of TEAEs (RR: 1.15 with 95% CI [1.06, 1.25], *P* = 0.0005) (moderate-quality evidence) (Fig. 5A, Table S3). The pooled studies were homogenous (*P* = 0.15, *I*^2^ = 41%). In network meta-analysis, OKZ 64 Q2w and Q4w regimens were significantly associated with a higher incidence of TEAEs (RR: 1.14 with 95% CI [1.03, 1.25]) and (RR: 1.15 with 95% CI [1.0,4 1.26]), respectively, with low observed heterogeneity of *I*^2^ = 34% (Table 3, Figs. S37-39).

#### Any TEAEs leading to drug discontinuation

In the pairwise meta-analysis, OKZ was significantly associated with more incidence of TEAEs leading to drug discontinuation (RR: 1.86 with 95% CI [1.05, 3.29], *P* = 0.03) (moderate-quality evidence) (Fig. 5B, Table S3). The pooled studies were homogenous (*P* = 0.34, *I*^2^ = 10%). In the network meta-analysis, OKZ 64 Q4w regimen was significantly associated with a higher incidence of TEAEs leading to drug discontinuation (RR: 2.03 with 95% CI [1.06, 3.89]), with no observed heterogeneity of *I*^2^ = 0% (Table 3, Figs. S40-42).

#### Any TESAEs

In the pairwise meta-analysis, we found no difference between OKZ and placebo regarding the incidence of TESAEs (RR: 1.12 with 95% CI [0.70, 1.78], *P* = 0.63) (moderate-quality evidence) (Fig. 4C, Table S3). The pooled studies were homogenous (*P* = 0.28, *I*^2^ = 21%). In the network meta-analysis, we found no difference between different OKZ regimens and placebo regarding the incidence of TESAEs, with low observed heterogeneity of *I*^2^ = 13% (Table 3, Figs. S43-45).

#### Any-cause mortality

In the pairwise meta-analysis, we found no difference between OKZ and placebo regarding the incidence of any-cause mortality (RR: 1.36 with 95% CI [0.23, 8.03], *P* = 0.74) (moderate-quality evidence) (Fig. 5D, Table S3). The pooled studies were homogenous (*P* = 0.94, *I*^2^ = 0%). In the network meta-analysis, we found no difference between different OKZ regimens and placebo regarding the incidence of any-cause mortality, with no observed heterogeneity of *I*^2^ = 0% (Table 3, Figs. S46-48).

#### Gastrointestinal disorders

In the pairwise meta-analysis, we found no difference between OKZ and placebo regarding the incidence of gastrointestinal disorders (RR: 1.20 with 95% CI [0.88, 1.64], *P* = 0.26) (moderate-quality evidence) (Fig. S46, Table S3). The pooled studies were homogenous (*P* = 0.84, *I*^2^ = 0%). In the network meta-analysis, we found no difference between different OKZ regimens and placebo regarding the incidence of gastrointestinal disorders, with no observed heterogeneity of *I*^2^ = 0% (Table 3, Figs. S50-52).

#### Infections

In the pairwise meta-analysis, we found no difference between OKZ and placebo regarding the incidence of infections (RR: 0.93 with 95% CI [0.79, 1.08], *P* = 0.34) (moderate-quality evidence) (Fig. S50, Table S3). The pooled studies were homogenous (*P* = 0.36, *I*^2^ = 8%). In the network meta-analysis, we found no difference between different OKZ regimens and placebo regarding the incidence of infections, with low observed heterogeneity of *I*^2^ = 12% (Table 3, Figs. S54-56).

## Discussion

This meta-analysis of five RCTs [15–19] found that the OKZ in RA patients with inadequate response to the standard of care was effective, compared to the placebo. All the disease activity indices favored OKZ except DAS28-ESR, which showed no difference from the placebo. The safety profile of the OKZ was as expected for the IL-6 inhibitors. TEAEs were higher in the OKZ group compared to the placebo. Also, the treatment discontinuation rate due to TEAEs was higher in the OKZ group. However, the OKZ and placebo groups were similar regarding all-cause mortality, TESAEs, gastrointestinal disorders, or infection.

OKZ is a newly developed humanized monoclonal antibody targeting IL-6 cytokine itself rather than its receptor. It has been investigated for the treatment of moderate to severe RA with inadequate response to TNF-α inhibitors in the presence of MTX. Only TCZ and sarilumab, which block the IL-6 receptor, have been approved for RA treatment (31,32). Moreover, other IL-6 inhibitors that target the IL-6 cytokine directly rather than its receptor (sirukuma, clazakizuma, and OKZ) are currently under development. However, none of them has been authorized yet for the treatment of RA. OKZ, being a direct IL-6 inhibitor, can be administrated less frequently compared to IL-6 receptor blockers as less protein dose is required to achieve an effect; hence, OKZ Q4W can be favored over weekly or biweekly dosing of the approved IL-6 receptor blockers (TCZ and sarilumab) [19].

ACR20 was used as the primary endpoint in this analysis because it has been widely accepted value for assessing the drugs’ efficacy in RA over the years. It also makes the comparison between the response to OKZ and other drugs that used the ACR20 value in the past reasonable [18]. Our pooled analysis showed significant improvement in the ACR20 in the OKZ group, compared to the placebo at 12 and 24 weeks following the treatment initiation. Furthermore, Smolen et al. [17] reported that the ACR20 improvement in patients treated with OKZ + MTX was similar to patients treated with adalimumab (TNF-α inhibitor) + MTX. Genovese et al. [15] also reported similar improvement in the ACR20 in patients treated with OKZ and TCZ (IL-6 receptor inhibitor). He also reported that the improvement in the ACR20 started as early as week 1 after treatment [15]. Similarly, ACR50 and ACR70 were significantly improved in the OKZ group versus placebo at weeks 12 and 24. ACR50 improvement was noticed as early as week 4 by Genovese et al. [15], and more patients met this index compared to the ACR70. ACR70 improvement was higher at 24 weeks, compared to 12 weeks indicating that longer treatment duration might induce more improvement.

Moreover, we found that the rate of DAS28-CRP ⩽ 3.2 was higher in the OKZ group versus the placebo. On the other hand, we did not find a difference between the two groups regarding DAS28-ESR. DAS28 index was first presented in 1995 using the count of 28 tender and swollen joints combined with a measure of the general health and acute phase reactant ESR. In 2004, CRP was suggested as an alternative component for the DAS28 instead of the ESR for different reasons. First, ESR is under the influence of different factors, including age, gender, and plasma proteins. Second, CRP is more susceptible to short-term alterations in inflammation, which in turn will reflect the rapid response to the treatment [15]. Since the development of the DAS28-CRP index, it has been investigated for validation and comparison to the original DAS28-ESR. Arguably, indices using CRP to evaluate drugs blocking IL-6 cannot be very accurate. This comes back to the fact that blocking IL-6 cytokine directly or blocking its receptor interferes with the production of CRP [17]. Therefore, due to possibly suspicious results, future studies should focus on clinical and radiological assessments. With debates on the superiority of one over the other, both indices have been validated by the EULAR and the ACR to monitor the disease severity and achieve treatment to the target concept [5]. To clarify, the DAS28 score ranges from 0 to 9.4, with values < 2.6 representing remission, while values ⩽ 3.2 represent low disease activity [5, 31].

Regarding CDAI, pooled data favored the OKZ over the placebo at weeks 12 and 24 of treatment. CDAI is another index to evaluate the disease severity in RA patients that does not use acute phase reactants for measurement. Instead, it uses the summation of the number of swollen/tender 28 joints plus patient and physician global assessment on the visual analog scale (VAS) [4]. The simplicity of this score measurement made it more feasible to be used in clinical settings. It has a score ranging from 0 to 76 where values ⩽ 2.8 represent remission, while values > 2.8 to 10 represent low disease activity [32]. CDAI has been validated compared to DAS28 and HAQ-DI indices [33] and recommended by the ACR to monitor the disease activity and achieve treatment to target [8, 34].

Regarding HAQ-DI, we found that patients’ disability improvement at week 12 based on HAQ-DI favored the OKZ group, compared to the placebo. Assessment of disability that results from either joint damage or pain in RA patients is important [35]. HAQ-DI is one of the most used patient-oriented tools to assess functional disability in RA patients. It has a range from 0 to 3, with higher numbers representing more disabilities [17]. The current consensus is that the lowest clinically significant difference in the HAQ-DI is a change of 0.22 to 0.25 [35]. However, Ward et al. suggested that a change of 0.375 is needed to show a clinically significant difference [35].

Moreover, OKZ safety profile was expected of IL-6 inhibitors and similar to the FDA-approved anti-IL-6 drugs, tocilizumab, and sarilumab [36, 37]. From the pooled data, we found that the OKZ group experienced more TEAEs and TEAEs leading to drug discontinuation compared to the placebo. However, we could not find a significant difference between the incidences of treatment-emergent any-cause mortality, TESAEs, gastrointestinal disorders, and infections between OKZ and placebo.

Genovese et al. [15] reported mild to moderate TEAEs in the OKZ group, which included gastrointestinal disorders, infections, and nervous system disorders. They found that TEAEs were similar in quality and frequency to those observed in the TCZ and placebo groups [15]. They also reported an increase in the patient’s liver enzymes in both the OKZ and TCZ groups without severe liver injury [15]. A mild increase in the patients’ lipids in both OKZ and TCZ was also noticed, besides a decrease in their neutrophilic counts [15]. Takeuchi et al. [16], Nasonov et al. [18], and Feist et al. [19] reported similar TEAE profiles, with most TEAEs being mild to moderate and more common in the OKZ groups. Furthermore, Smolen et al. [17] reported that most of the TEAEs were mild to moderate as well and were similar in all treatment groups, including the adalimumab group.

Higher doses or higher frequency of OKZ administration are likely to cause higher TEAEs or TESAEs. This was supported by Feist et al. [19] reporting a frequency-dependent increase in the rate of TESAEs in most of the events in the OKZ 64 mg once every two weeks (Q2W) group. Genovese et al. [15] also reported similar findings as they noticed higher injection site reactions in the OKZ 240 mg Q2W group. However, after we analyzed the pooled data, we found that OKZ 64 mg Q2W and Q4W were both associated with a higher rate TEAEs, compared to the placebo. Also, our network meta-analysis found that drug discontinuation due to TEAEs was higher in the OKZ 64 mg Q2W group. Based on our observations, the concept of a dose-dependent increase in TEAEs or TESAEs with OKZ might need further research.

With most of the reported TEAEs being mild to moderate in all the included studies, few TESAEs were reported. Two TESAEs were reported by Genovese et al. [15], one pneumonia and another abscess in each of the OKZ and TCZ groups. Takeuchi et al. [16] also reported four TESAEs, including two RAs in the placebo group and two infections in the OKZ group. Nasonov et al. [18] reported few TESAEs in the OKZ groups, including pulmonary tuberculosis. The largest number of the TESAEs were reported by Smolen et al. [17], including major cardiovascular events (which were similar in the OKZ and adalimumab groups), pneumonia, sepsis, and pulmonary tuberculosis. The reported TESAEs by Feist et al. [19] included sepsis, hepatobiliary disorders, and cellulitis.

Furthermore, no deaths were reported by Genovese et al. [15], Takeuchi et al. [16], or Feist et al. [19]. However, Nasonov et al. [18] reported one death in the OKZ 64 mg Q2W group, and Smolen et al. [17] also reported deaths due to TESEAs in three patients in the OKZ 64 mg Q2W group, two patients in the OKZ 64 mg Q4W group, one patient in the adalimumab group, and one patient receiving placebo.

## Strengths

This is the first meta-analysis, to the best of our knowledge, that evaluated the efficacy and safety of OKZ for RA, investigating the optimal regimen through a thorough network meta-analysis. Our review was strictly conducted according to PRISMA guidelines [20] with prospectively published protocol and GRADE evidence assessment.

## Limitations

Our review is limited by the following: first, some of our network meta-analysis comparisons included only one to two arms, and our 24-week pairwise analysis included only two RCTs, which can limit the external validity of its findings. Second, the number of patients in the OKZ group is relatively higher than the placebo group, which is an inherited limitation of the included trials due to ethical reasons. Third, none of the included trials conducted a radiological examination of the joints to confirm the observed effects. Finally, some of our meta-analysis outcomes showed significant heterogeneity.

## Implications for future research

By combining the data from three phase III and two phase II RCTs, we gained more power to provide the highest level of evidence to estimate the effectiveness and safety of OKZ as a new treatment for RA. Our study confirmed the added treatment benefits of OKZ when combined with MTX. The lack of differences in TESAEs and any-cause mortality between different doses of OKZ and placebo confirmed the safety profile of this new medication. Our study justifies the need for designing more RCTs investigating, the still-lacking, long-term effects and side effects of OKZ beyond 24 weeks. It also points to the need to investigate the most effective dose of OKZ, given the dose–response increase in TEAEs incidence. With most of the included studies comparing the OKZ to placebo, the need to look into the comparative efficacy of OKZ versus the established TNF-α inhibitors and IL-6 inhibitors became important. Eventually, the results of this study might provide supportive evidence for getting the OKZ approved as a treatment for RA. A step equips physicians with a new tool for fighting this disease and gives hope to many RA patients.

## Conclusion

OKZ treatment with MTX was effective in improving RA indices with an improvement of the RA’s symptoms and signs along with the expected safety profile; however, more large-scale RCTs are still required to investigate the optimal dosing, long-term effects, and comparative efficacy versus established biological DMARDs.

## Supplementary Information

Below is the link to the electronic supplementary material.

Supplementary file1 (DOCX 3460 KB)

## Data Availability

Not applicable.
