# Supplementary material for: The efficacy and safety of olokizumab for rheumatoid arthritis: a systematic review, pairwise, and network meta-analysis
Source: Clin Rheumatol. 2023 Feb 16;42(6):1503–20. doi: 10.1007/s10067-023-06519-6 (PMC10202974; doi:10.1007/s10067-023-06519-6)
Supplement: Supplementary file 1 — Supplementary file1 (DOCX 3460 KB) [file 10067_2023_6519_MOESM1_ESM.docx]

**Title.**

**The Efficacy and Safety of Olokizumab for Rheumatoid Arthritis: A Systematic Review, Pairwise, and Network Meta-Analysis.**

**Running Title.**

Olokizumab for Rheumatoid Arthritis

**Authors.**

Mohamed Abuelazm^1,*^, Ahmed Ghanem^2,*^, Abdelrahman Mahmoud^3^, Aml Brakat^4^, Mohamad A. Elzeftawy^1^, Aya Fayoud^5^, Ahmed K. Awad^6^, Basel Abdelazeem^7.8^

**Affiliations.**

1. Faculty of Medicine, Tanta University, Tanta, Egypt.
2. Cardiology Department, The Lundquist Institute, Torrance, CA, USA.
3. Faculty of Medicine, Minia University, Minia, Egypt.
4. Faculty of Medicine, Zagazig University, Zagazig, Egypt.
5. Faculty of Pharmacy, Kafr el-shiekh University, Kafr el-shiekh, Egypt.
6. Faculty of Medicine, Ain-Shams University, Cairo, Egypt.
7. Department of Internal Medicine, McLaren Health Care, Flint, Michigan, USA.
8. Department of Internal Medicine, Michigan State University, East Lansing, Michigan.

*, Both authors contributed equally to this work.

**Keywords.**

Olokizumab; OKZ; rheumatoid arthritis; DMARDs; systematic review; meta-analysis.

**Corresponding author.**

**Contents:**

**Tables.**

Table S1: PRISMA 2020 checklist.

Table S2: Search terms and results in different databases.

Table S3: GRADE evidence profile.

Table S4: Sensitivity analysis of the efficacy outcomes.

**Figures.**

Figure S1: Forest plot of pooled summary estimates derived from network meta-analysis for ACR20 response after 12 weeks.

Figure S2: Network plot of ACR20 response after 12 weeks.

Figure S3: Forest plot of individual study results grouped by treatment component for ACR20 response after 12 weeks.

Figure S4: Forest plot of pooled summary estimates derived from network meta-analysis for ACR20 response after 24 weeks.

Figure S5: Network plot of ACR20 response after 24 weeks.

Figure S6: Forest plot of individual study results grouped by treatment component for ACR20 response after 24 weeks.

Figure S7: Forest plot of pooled summary estimates derived from network meta-analysis for ACR50 response after 12 weeks.

Figure S8: Network plot of ACR50 response after 12 weeks.

Figure S9: Forest plot of individual study results grouped by treatment component for ACR50 response after 12 weeks.

Figure S10: Forest plot of pooled summary estimates derived from network meta-analysis for ACR50 response after 24 weeks.

Figure S11: Network plot of ACR50 response after 24 weeks.

Figure S12: Forest plot of individual study results grouped by treatment component for ACR50 response after 24 weeks.

Figure S13: Forest plot of pooled summary estimates derived from network meta-analysis for ACR70 response after 12 weeks.

Figure S14: Network plot of ACR70 response after 12 weeks.

Figure S15: Forest plot of individual study results grouped by treatment component for ACR70 response after 12 weeks.

Figure S16: Forest plot of pooled summary estimates derived from network meta-analysis for ACR70 response after 24 weeks.

Figure S17: Network plot of ACR70 response after 24 weeks.

Figure S18: Forest plot of individual study results grouped by treatment component for DAS28-CRP <3.2 after 24 weeks.

Figure S19: Forest plot of pooled summary estimates derived from network meta-analysis for DAS28-CRP <3.2 after 12 weeks.

Figure S20: Network plot of DAS28-CRP <3.2 after 12 weeks.

Figure S21: Forest plot of individual study results grouped by treatment component for DAS28-CRP <3.2 after 12 weeks.

Figure S22: Forest plot of pooled summary estimates derived from network meta-analysis for DAS28-CRP <3.2 after 24 weeks.

Figure S23: Network plot of DAS28-CRP <3.2 after 24 weeks.

Figure S24: Forest plot of individual study results grouped by treatment component for DAS28-CRP <3.2 after 24 weeks.

Figure S25: Forest plot of pair-wise analysis of CDAI Score of ≤2.8.

Figure S26: Forest plot of pooled summary estimates derived from network meta-analysis for CDAI Score of ≤2.8 after 12 weeks.

Figure S27: Network plot of CDAI Score of ≤2.8 after 12 weeks.

Figure S28: Forest plot of individual study results grouped by treatment component for CDAI Score of ≤2.8 after 12 weeks.

Figure S29: Forest plot of pooled summary estimates derived from network meta-analysis for CDAI Score of ≤2.8 after 24 weeks.

Figure S30: Network plot of CDAI Score of ≤2.8 after 24 weeks.

Figure S31: Forest plot of individual study results grouped by treatment component for CDAI Score of ≤2.8 after 24 weeks.

Figure S32: Forest plot of pair-wise analysis of HAQ-DI score change after 12 weeks.

Figure S33: Forest plot of pooled summary estimates derived from network meta-analysis for HAQ-DI score change after 12 weeks.

Figure S34: Network plot of HAQ-DI score change after 12 weeks.

Figure S35: Forest plot of individual study results grouped by treatment component for HAQ-DI score change after 12 weeks.

Figure S36: Forest plot of pair-wise analysis of DAS28-ESR score change after 12 weeks.

Figure S37: Forest plot of pooled summary estimates derived from network meta-analysis for any TEAEs.

Figure S38: Network plot of any TEAEs.

Figure S39: Forest plot of individual study results grouped by treatment component for any TEAEs.

Figure S40: Forest plot of pooled summary estimates derived from network meta-analysis for any TEAEs leading to drug discontinuation.

Figure S41: Network plot of any TEAEs leading to drug discontinuation.

Figure S42: Forest plot of individual study results grouped by treatment component any TEAEs leading to drug discontinuation.

Figure S43: Forest plot of pooled summary estimates derived from network meta-analysis for any TESAEs.

Figure S44: Network plot of any TESAEs.

Figure S45: Forest plot of individual study results grouped by treatment component for any TESAEs.

Figure S46: Forest plot of pooled summary estimates derived from network meta-analysis for any-cause mortality.

Figure S47: Network plot of any-cause mortality.

Figure S48: Forest plot of individual study results grouped by treatment component for any-cause mortality.

Figure S49: Forest plot of pair-wise analysis of gastrointestinal disorders.

Figure S50: Forest plot of pooled summary estimates derived from network meta-analysis for gastrointestinal disorders.

Figure S51: Network plot of gastrointestinal disorders.

Figure S52: Forest plot of individual study results grouped by treatment component for gastrointestinal disorders.

Figure S53: Forest plot of pair-wise analysis of infections.

Figure S54: Forest plot of pooled summary estimates derived from network meta-analysis for infections.

Figure S55: Network plot of infections.

Figure S56: Forest plot of individual study results grouped by treatment component for infections.

| **Section and Topic** | **Item #** | **Checklist item** | **Location where item is reported** |
| --- | --- | --- | --- |
| **TITLE** | | |  |
| Title | 1 | Identify the report as a systematic review. | Line 2 |
| **ABSTRACT** | | |  |
| Abstract | 2 | See the PRISMA 2020 for Abstracts checklist. | Page 2 |
| **INTRODUCTION** | | |  |
| Rationale | 3 | Describe the rationale for the review in the context of existing knowledge. | Page 5 |
| Objectives | 4 | Provide an explicit statement of the objective(s) or question(s) the review addresses. | Page 5 |
| **METHODS** | | |  |
| Eligibility criteria | 5 | Specify the inclusion and exclusion criteria for the review and how studies were grouped for the syntheses. | Page 6, subsection 2.3 |
| Information sources | 6 | Specify all databases, registers, websites, organisations, reference lists and other sources searched or consulted to identify studies. Specify the date when each source was last searched or consulted. | Page 6, subsection 2.2 |
| Search strategy | 7 | Present the full search strategies for all databases, registers and websites, including any filters and limits used. | Supplementary material, table S2 |
| Selection process | 8 | Specify the methods used to decide whether a study met the inclusion criteria of the review, including how many reviewers screened each record and each report retrieved, whether they worked independently, and if applicable, details of automation tools used in the process. | Page 7, subsection 2.4 |
| Data collection process | 9 | Specify the methods used to collect data from reports, including how many reviewers collected data from each report, whether they worked independently, any processes for obtaining or confirming data from study investigators, and if applicable, details of automation tools used in the process. | Page 7, subsection 2.5 |
| Data items | 10a | List and define all outcomes for which data were sought. Specify whether all results that were compatible with each outcome domain in each study were sought (e.g. for all measures, time points, analyses), and if not, the methods used to decide which results to collect. | Page 7, subsection 2.5 |
|  | 10b | List and define all other variables for which data were sought (e.g. participant and intervention characteristics, funding sources). Describe any assumptions made about any missing or unclear information. | Page 7, subsection 2.5 |
| Study risk of bias assessment | 11 | Specify the methods used to assess risk of bias in the included studies, including details of the tool(s) used, how many reviewers assessed each study and whether they worked independently, and if applicable, details of automation tools used in the process. | Page 7, subsection 2.6 |
| Effect measures | 12 | Specify for each outcome the effect measure(s) (e.g. risk ratio, mean difference) used in the synthesis or presentation of results. | Page 8, subsection 2.7 |
| Synthesis methods | 13a | Describe the processes used to decide which studies were eligible for each synthesis (e.g. tabulating the study intervention characteristics and comparing against the planned groups for each synthesis (item #5)). | Page 8, subsection 2.7 |
|  | 13b | Describe any methods required to prepare the data for presentation or synthesis, such as handling of missing summary statistics, or data conversions. | Page 8, subsection 2.7 |
|  | 13c | Describe any methods used to tabulate or visually display results of individual studies and syntheses. | Page 8, subsection 2.7 |
|  | 13d | Describe any methods used to synthesize results and provide a rationale for the choice(s). If meta-analysis was performed, describe the model(s), method(s) to identify the presence and extent of statistical heterogeneity, and software package(s) used. | Page 8, subsection 2.7 |
|  | 13e | Describe any methods used to explore possible causes of heterogeneity among study results (e.g. subgroup analysis, meta-regression). | Page 8, subsection 2.7 |
|  | 13f | Describe any sensitivity analyses conducted to assess robustness of the synthesized results. | Page 8, subsection 2.7 |
| Reporting bias assessment | 14 | Describe any methods used to assess risk of bias due to missing results in a synthesis (arising from reporting biases). | Page 7, subsection 2.6 |
| Certainty assessment | 15 | Describe any methods used to assess certainty (or confidence) in the body of evidence for an outcome. | Page 7, subsection 2.6 |
| **RESULTS** | | |  |
| Study selection | 16a | Describe the results of the search and selection process, from the number of records identified in the search to the number of studies included in the review, ideally using a flow diagram. | Page 9, subsection 3.1 |
|  | 16b | Cite studies that might appear to meet the inclusion criteria, but which were excluded, and explain why they were excluded. | Not applicable |
| Study characteristics | 17 | Cite each included study and present its characteristics. | Page 9, subsection 3.2 |
| Risk of bias in studies | 18 | Present assessments of risk of bias for each included study. | Page 9, subsection 3.3 |
| Results of individual studies | 19 | For all outcomes, present, for each study: (a) summary statistics for each group (where appropriate) and (b) an effect estimate and its precision (e.g. confidence/credible interval), ideally using structured tables or plots. | Pages 10-13, subsections 3.4-3.5 |
| Results of syntheses | 20a | For each synthesis, briefly summarise the characteristics and risk of bias among contributing studies. | Page 9, subsection 3.3 |
|  | 20b | Present results of all statistical syntheses conducted. If meta-analysis was done, present for each the summary estimate and its precision (e.g. confidence/credible interval) and measures of statistical heterogeneity. If comparing groups, describe the direction of the effect. | Pages 10-13, subsections 3.4-3.5 |
|  | 20c | Present results of all investigations of possible causes of heterogeneity among study results. | Pages 10-13, subsections 3.4-3.5 |
|  | 20d | Present results of all sensitivity analyses conducted to assess the robustness of the synthesized results. | Not applicable |
| Reporting biases | 21 | Present assessments of risk of bias due to missing results (arising from reporting biases) for each synthesis assessed. | Table S4 |
| Certainty of evidence | 22 | Present assessments of certainty (or confidence) in the body of evidence for each outcome assessed. | Table S3 |
| **DISCUSSION** | | |  |
| Discussion | 23a | Provide a general interpretation of the results in the context of other evidence. | Page 19 |
|  | 23b | Discuss any limitations of the evidence included in the review. | Page 18 |
|  | 23c | Discuss any limitations of the review processes used. | Page 18 |
|  | 23d | Discuss implications of the results for practice, policy, and future research. | Page 18 |
| **OTHER INFORMATION** | | |  |
| Registration and protocol | 24a | Provide registration information for the review, including register name and registration number, or state that the review was not registered. | Page 6, subsection 2.1 |
|  | 24b | Indicate where the review protocol can be accessed, or state that a protocol was not prepared. | Page 6, subsection 2.1 |
|  | 24c | Describe and explain any amendments to information provided at registration or in the protocol. | Page 6, subsection 2.1 |
| Support | 25 | Describe sources of financial or non-financial support for the review, and the role of the funders or sponsors in the review. | Page 20 |
| Competing interests | 26 | Declare any competing interests of review authors. | Page 19 |
| Availability of data, code and other materials | 27 | Report which of the following are publicly available and where they can be found: template data collection forms; data extracted from included studies; data used for all analyses; analytic code; any other materials used in the review. | Page 19 |

Table S1 PRISMA 2020 checklist

| Database | Search Terms | Search Field | Search Results |
| --- | --- | --- | --- |
| Pubmed | (Olokizumab OR CDP-6038 OR CDP6038) AND ("Rheumatoid arthritis" OR "arthritis Rheumatoid" OR RA) | All Field | 18 |
| Cochrane | (Olokizumab OR CDP-6038 OR CDP6038) AND ("Rheumatoid arthritis" OR "arthritis Rheumatoid" OR RA) | All Field | 36 |
| WOS | (Olokizumab OR CDP-6038 OR CDP6038) AND ("Rheumatoid arthritis" OR "arthritis Rheumatoid" OR RA) | All Field | 37 |
| SCOPUS | TITLE-ABS-KEY (( olokizumab  OR  cdp-6038  OR  cdp6038 )  AND  ( "Rheumatoid arthritis"  OR  "arthritis Rheumatoid"  OR  RA )) | Title, Abstract, Keywords | 76 |
| EMBASE | #3.  #1 AND #2               34  #2.  'rheumatoid arthritis':ti,ab,kw OR 'arthritis       rheumatoid':ti,ab,kw OR ra:ti,ab,kw 249,164  #1.  olokizumab:ti,ab,kw OR 'cdp 6038':ti,ab,kw OR cdp6038:ti,ab,kw 52 | All Field | 34 |

Table S2. Search terms and results in different databases.

| **Certainty assessment** | | | | | | | **№ of patients** | | **Effect** | | **Certainty** |
| --- | --- | --- | --- | --- | --- | --- | --- | --- | --- | --- | --- |
| **№ of studies** | **Study design** | **Risk of bias** | **Inconsistency** | **Indirectness** | **Imprecision** | **Other considerations** | **[intervention]** | **[comparison]** | **Relative (95% CI)** | **Absolute (95% CI)** |  |
| **DAS28-CRP <3.2 - After 12 Weeks** | | | | | | | | | | | |
| 5 | randomised trials | not serious | not serious | not serious | not serious | none | 714/1749 (40.8%) | 52/528 (9.8%) | **RR 3.91** (2.65 to 5.79) | **287 more per 1,000** (from 162 more to 472 more) | ⨁⨁⨁⨁ High |
| **DAS28-CRP <3.2 - After 24 Weeks** | | | | | | | | | | | |
| 2 | randomised trials | not serious | serious^a^ | not serious | serious^b^ | none | 623/1228 (50.7%) | 64/386 (16.6%) | **RR 3.54** (1.55 to 8.10) | **421 more per 1,000** (from 91 more to 1,000 more) | ⨁⨁◯◯ Low |
| **CDAI Score <2.8 - After 12 Weeks** | | | | | | | | | | | |
| 3 | randomised trials | not serious | not serious | not serious | serious^c^ | none | 93/1527 (6.1%) | 8/455 (1.8%) | **RR 2.80** (1.43 to 5.48) | **32 more per 1,000** (from 8 more to 79 more) | ⨁⨁⨁◯ Moderate |
| **CDAI Score <2.8 - After 24 Weeks** | | | | | | | | | | | |
| 2 | randomised trials | not serious | serious^a^ | not serious | serious^c^ | none | 133/1228 (10.8%) | 10/386 (2.6%) | **RR 3.67** (2.01 to 6.72) | **69 more per 1,000** (from 26 more to 148 more) | ⨁⨁◯◯ Low |
| **HAQ-DI Change from baseline after 12 weeks** | | | | | | | | | | | |
| 5 | randomised trials | not serious | serious^a^ | not serious | not serious | none | 1749 | 528 | - | MD **0.28 lower** (0.38 lower to 0.18 lower) | ⨁⨁⨁◯ Moderate |
| **DAS28 (ESR) Change from baseline after 12 weeks** | | | | | | | | | | | |
| 2 | randomised trials | not serious | very serious^d^ | not serious | very serious^b^ | none | 222 | 73 | - | MD **3.69 lower** (8.13 lower to 0.75 higher) | ⨁◯◯◯ Very low |
| **ACR20 - After 12 Weeks** | | | | | | | | | | | |
| 5 | randomised trials | not serious | serious^a^ | not serious | not serious | none | 1152/1749 (65.9%) | 187/528 (35.4%) | **RR 1.97** (1.49 to 2.58) | **344 more per 1,000** (from 174 more to 560 more) | ⨁⨁⨁◯ Moderate |
| **ACR20 - After 24 Weeks** | | | | | | | | | | | |
| 2 | randomised trials | not serious | serious^a^ | not serious | not serious | none | 885/1228 (72.1%) | 162/386 (42.0%) | **RR 1.75** (1.35 to 2.27) | **315 more per 1,000** (from 147 more to 533 more) | ⨁⨁⨁◯ Moderate |
| **ACR50 - After 12 Weeks** | | | | | | | | | | | |
| 5 | randomised trials | not serious | serious^a^ | not serious | not serious | none | 660/1749 (37.7%) | 58/528 (11.0%) | **RR 3.83** (2.13 to 6.87) | **311 more per 1,000** (from 124 more to 645 more) | ⨁⨁⨁◯ Moderate |
| **ACR50 - After 24 Weeks** | | | | | | | | | | | |
| 2 | randomised trials | not serious | serious^a^ | not serious | serious^b^ | none | 613/1228 (49.9%) | 66/386 (17.1%) | **RR 3.53** (1.35 to 9.23) | **433 more per 1,000** (from 60 more to 1,000 more) | ⨁⨁◯◯ Low |
| **ACR70 - After 12 Weeks** | | | | | | | | | | | |
| 3 | randomised trials | not serious | not serious | not serious | serious^b^ | none | 101/674 (15.0%) | 6/241 (2.5%) | **RR 5.09** (1.53 to 16.91) | **102 more per 1,000** (from 13 more to 396 more) | ⨁⨁⨁◯ Moderate |
| **ACR70 - After 24 Weeks** | | | | | | | | | | | |
| 2 | randomised trials | not serious | serious^a^ | not serious | serious^b^ | none | 322/1228 (26.2%) | 30/386 (7.8%) | **RR 4.52** (1.13 to 18.02) | **274 more per 1,000** (from 10 more to 1,000 more) | ⨁⨁◯◯ Low |
| **Any Treatment Emergent Adverse Events (TAEAs)** | | | | | | | | | | | |
| 5 | randomised trials | not serious | not serious | not serious | serious^e^ | none | 1164/1746 (66.7%) | 301/528 (57.0%) | **RR 1.15** (1.06 to 1.25) | **86 more per 1,000** (from 34 more to 143 more) | ⨁⨁⨁◯ Moderate |
| **Any Serious TAEAs** | | | | | | | | | | | |
| 5 | randomised trials | not serious | not serious | not serious | serious^e^ | none | 78/1746 (4.5%) | 21/528 (4.0%) | **RR 1.12** (0.70 to 1.78) | **5 more per 1,000** (from 12 fewer to 31 more) | ⨁⨁⨁◯ Moderate |
| **All-Cause Mortality** | | | | | | | | | | | |
| 5 | randomised trials | not serious | not serious | not serious | serious^e^ | none | 6/1746 (0.3%) | 1/528 (0.2%) | **RR 1.36** (0.23 to 8.03) | **1 more per 1,000** (from 1 fewer to 13 more) | ⨁⨁⨁◯ Moderate |
| **Adverse event leading to discontinuation of OKZ** | | | | | | | | | | | |
| 4 | randomised trials | not serious | not serious | not serious | serious^e^ | none | 85/1614 (5.3%) | 13/484 (2.7%) | **RR 1.86** (1.05 to 3.29) | **23 more per 1,000** (from 1 more to 62 more) | ⨁⨁⨁◯ Moderate |
| **Gastrointestinal Disorder** | | | | | | | | | | | |
| 5 | randomised trials | not serious | not serious | not serious | serious^e^ | none | 184/1746 (10.5%) | 42/528 (8.0%) | **RR 1.20** (0.88 to 1.64) | **16 more per 1,000** (from 10 fewer to 51 more) | ⨁⨁⨁◯ Moderate |
| **Infection** | | | | | | | | | | | |
| 5 | randomised trials | not serious | not serious | not serious | serious^e^ | none | 482/1746 (27.6%) | 149/528 (28.2%) | **RR 0.93** (0.79 to 1.08) | **20 fewer per 1,000** (from 59 fewer to 23 more) | ⨁⨁⨁◯ Moderate |

Table S3: GRADE evidence profile.

CI: confidence interval; MD: mean difference; RR: risk ratio

**Explanations:**

a. I^2^ test > 50%.

b. Wide confidence interval.

c. Number of events is fewer than 300 events.

d. I^2^ test > 90%.

e. Confidence interval does not exclude the risk of appreciable benefit/harm

| Outcome | Number of  participants (OKZ/Placebo) | No. of  trials | Quantitative data synthesis | | | | Heterogeneity analysis | | |
| --- | --- | --- | --- | --- | --- | --- | --- | --- | --- |
|  |  |  | RR/MD | 95% CI | Z value | p-value | df | p-value | I2 (%) |
| **ACR20 Response after 12 weeks** | | | | | | | | | |
| All studies | 1749/528 | 5 | 1.97 | [1.49, 2.58] | 4.83 | 0.00001 | 4 | 0.01 | 70 |
| Omitting  Feist et al. 2022 | 1450/459 | 4 | 2.19 | [1.52, 3.14] | 4.21 | 0.0001 | 3 | 0.008 | 75 |
| Omitting  Genovese et al. 2014 | 1617/484 | 4 | 1.92 | [1.42, 2.60] | 4.25 | 0.0001 | 3 | 0.007 | 75 |
| Omitting  Nasonov et al. 2021 | 1464/385 | 4 | 1.70 | [1.37, 2.10] | 4.88 | 0.00001 | 3 | 0.21 | 34 |
| Omitting  Smolen et al. 2022 | 806/285 | 4 | 2.17 | [1.51, 3.13] | 4.16 | 0.0001 | 3 | 0.04 | 64 |
| Omitting  Takeuchi et al. 2016 | 1659/499 | 4 | 1.87 | [1.42, 2.48] | 4.39 | 0.0001 | 3 | 0.01 | 72 |
| **ACR20 Response after 24 weeks** | | | | | | | | | |
| All studies | 1749/528 | 5 | 3.83 | [2.13, 6.87] | 4.5 | 0.00001 | 4 | 0.02 | 67 |
| Omitting  Feist et al. 2022 | 1450/459 | 4 | 5.07 | [2.22, 11.60] | 3.85 | 0.0001 | 3 | 0.02 | 70 |
| Omitting  Genovese et al. 2014 | 1617/484 | 4 | 3.61 | [1.96, 6.66] | 4.12 | 0.0001 | 3 | 0.01 | 72 |
| Omitting  Nasonov et al. 2021 | 1464/385 | 4 | 2.66 | [1.98, 3.59] | 6.43 | 0.00001 | 3 | 0.36 | 7 |
| Omitting  Smolen et al. 2022 | 806/285 | 4 | 4.87 | [1.86, 12.78] | 3.22 | 0.001 | 3 | 0.01 | 72 |
| Omitting  Takeuchi et al. 2016 | 1659/499 | 4 | 3.68 | [1.92, 7.06] | 3.92 | 0.0001 | 3 | 0.01 | 73 |
| **HAQ-DI Score Change after 12 weeks** | | | | | | | | | |
| All studies | 1749/528 | 5 | -0.28 | [-0.38, -0.18] | 5.32 | 0.00001 | 4 | 0.002 | 76 |
| Omitting  Feist et al. 2022 | 1450/459 | 4 | -0.32 | [-0.45, -0.20] | 5.04 | 0.00001 | 3 | 0.001 | 81 |
| Omitting  Genovese et al. 2014 | 1617/484 | 4 | -0.26 | [-0.36, -0.15] | 4.81 | 0.00001 | 3 | 0.005 | 76 |
| Omitting  Nasonov et al. 2021 | 1464/385 | 4 | -0.26 | [-0.37, -0.14] | 4.32 | 0.0001 | 3 | 0.03 | 67 |
| Omitting  Smolen et al. 2022 | 806/285 | 4 | -0.31 | [-0.45, -0.18] | 4.46 | 0.00001 | 3 | 0.03 | 66 |
| Omitting  Takeuchi et al. 2016 | 1659/499 | 4 | -0.26 | [-0.37, -0.15] | 4.73 | 0.00001 | 3 | 0.003 | 78 |

Table S4 Sensitivity analysis of the efficacy outcomes.

CI: confidence interval; df: degrees of freedom; MD: mean difference.


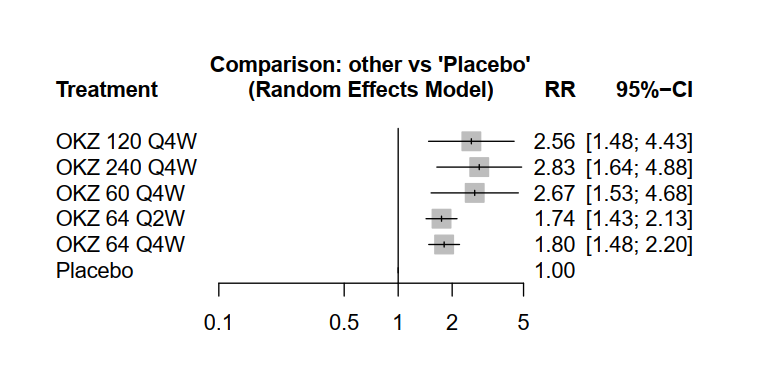


Figure S1: Forest plot of pooled summary estimates derived from network meta-analysis for ACR20 response after 12 weeks.


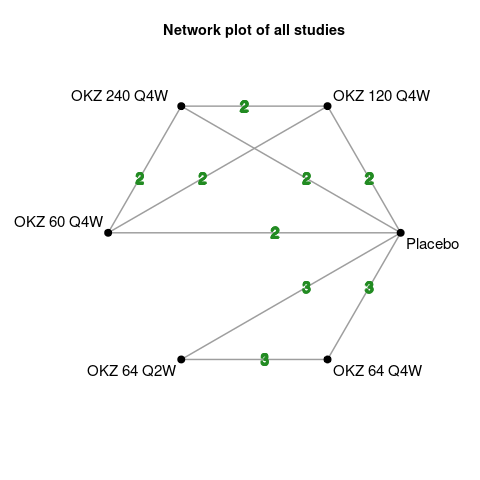


Figure S2: Network plot of ACR20 response after 12 weeks.


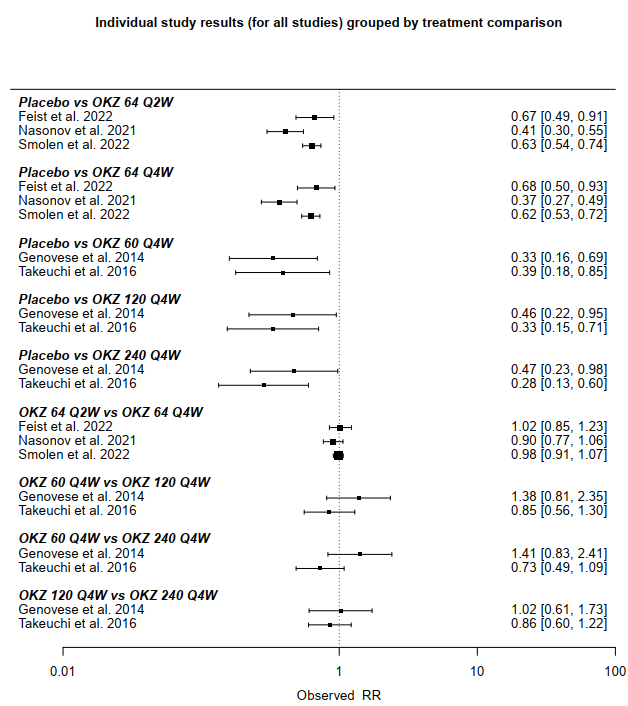


Figure S3: Forest plot of individual study results grouped by treatment component for ACR20 response after 12 weeks.


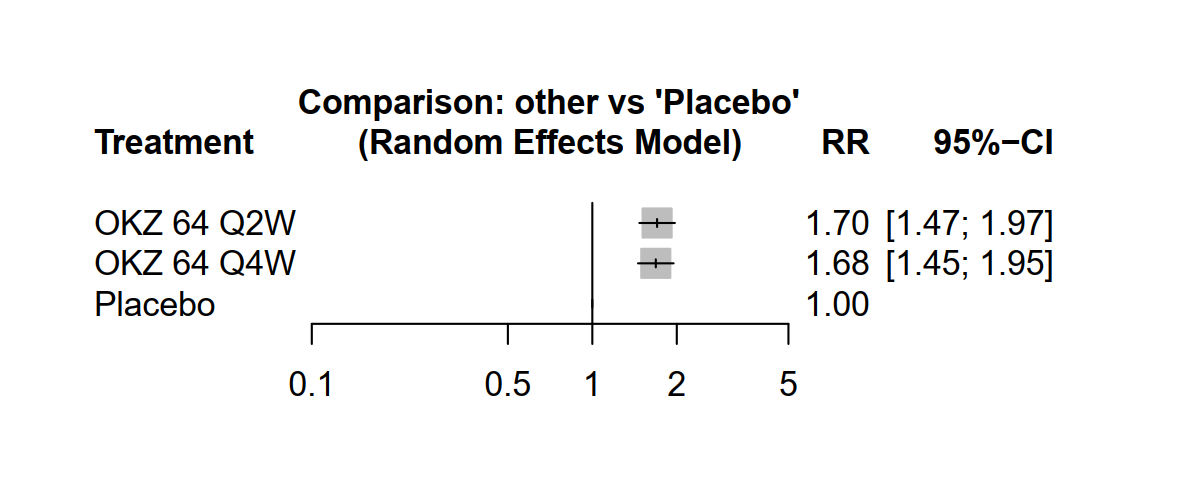


Figure S4: Forest plot of pooled summary estimates derived from network meta-analysis for ACR20 response after 24 weeks.


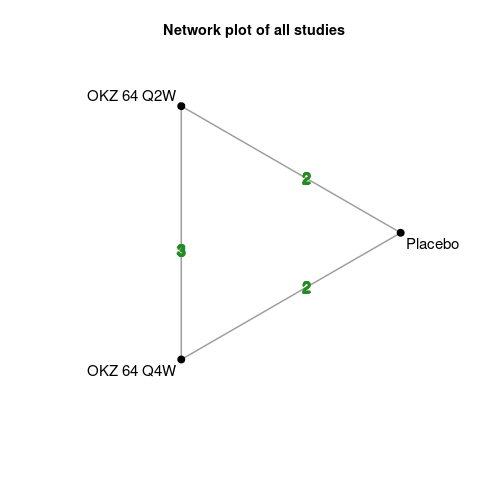


Figure S5: Network plot of ACR20 response after 24 weeks.


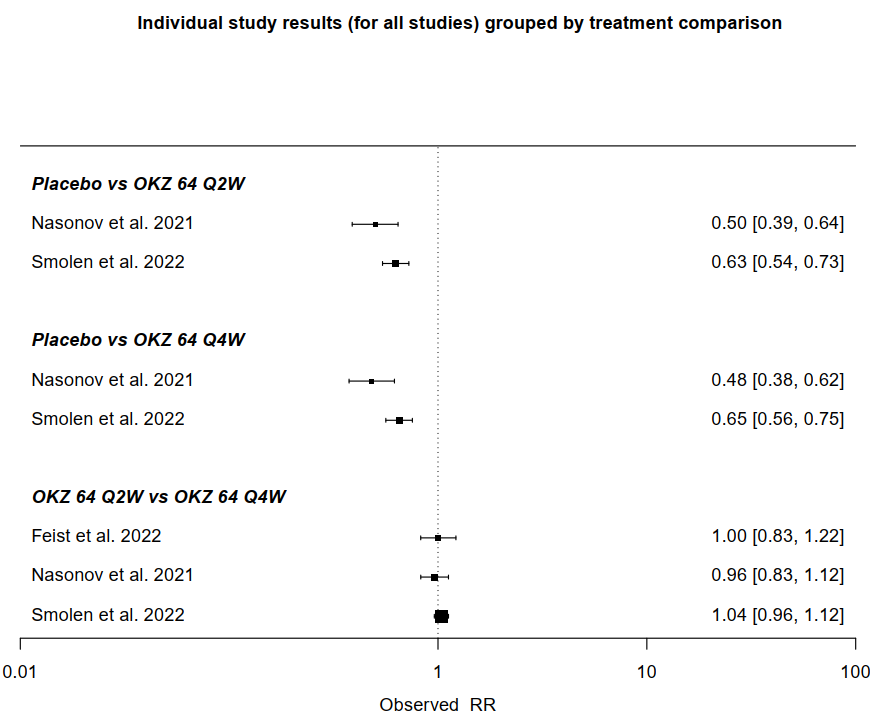


Figure S6: Forest plot of individual study results grouped by treatment component for ACR20 response after 24 weeks.


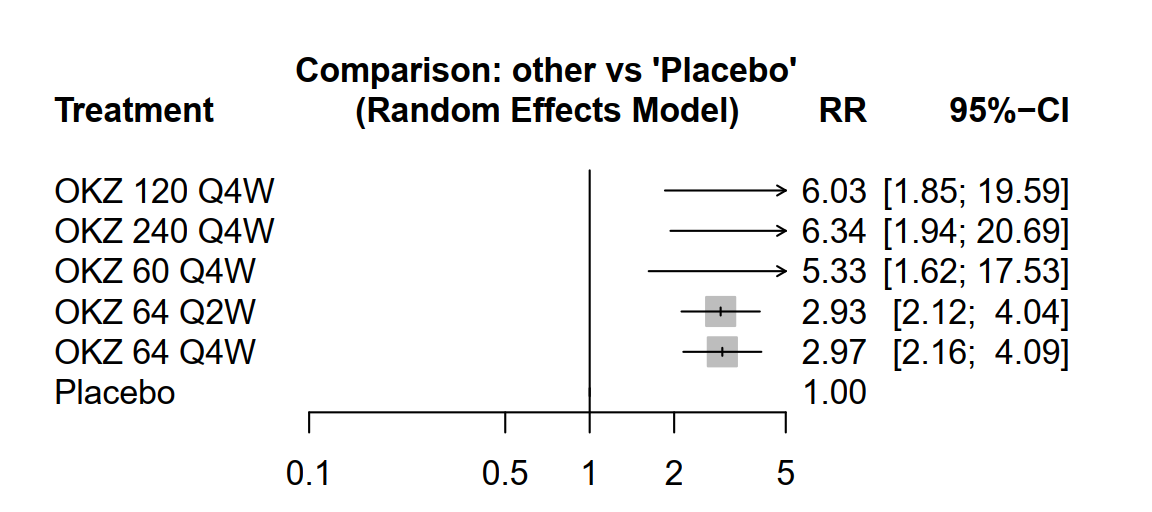


Figure S7: Forest plot of pooled summary estimates derived from network meta-analysis for ACR50 response after 12 weeks.


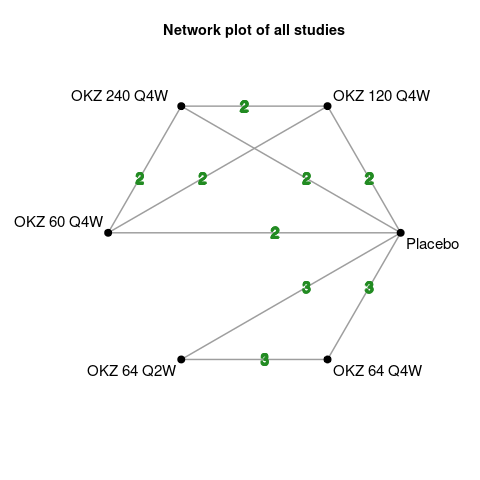


Figure S8:Network plot of ACR50 response after 12 weeks.


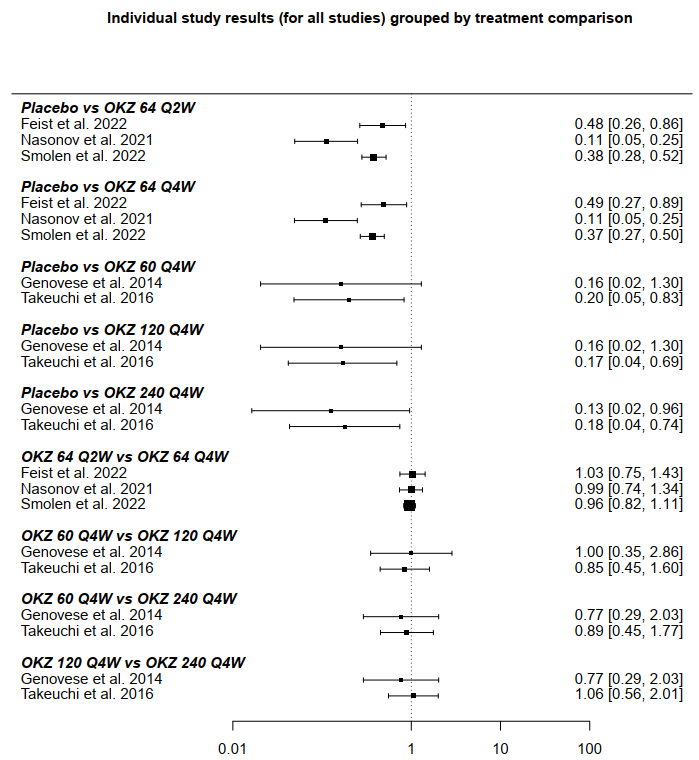


Figure S9: Forest plot of individual study results grouped by treatment component for ACR50 response after 12 weeks.


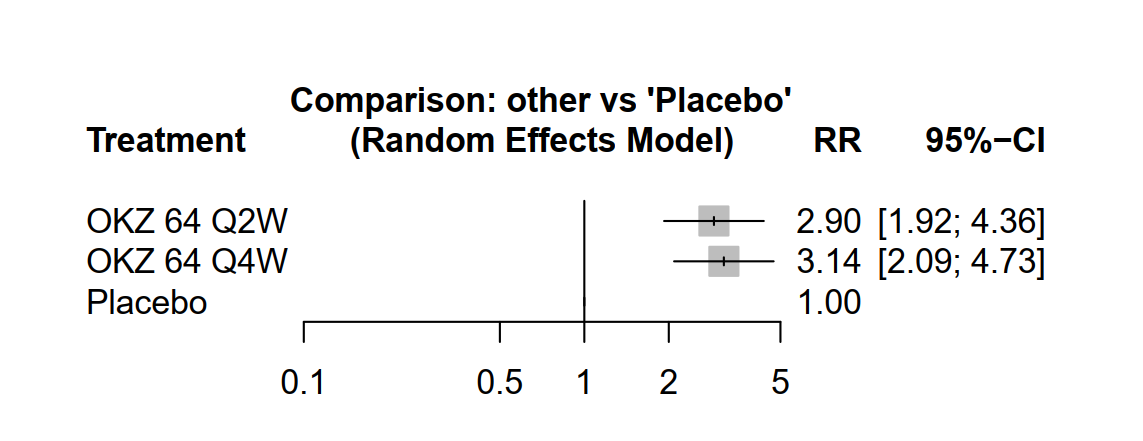


Figure S10: Forest plot of pooled summary estimates derived from network meta-analysis for ACR50 response after 24 weeks.


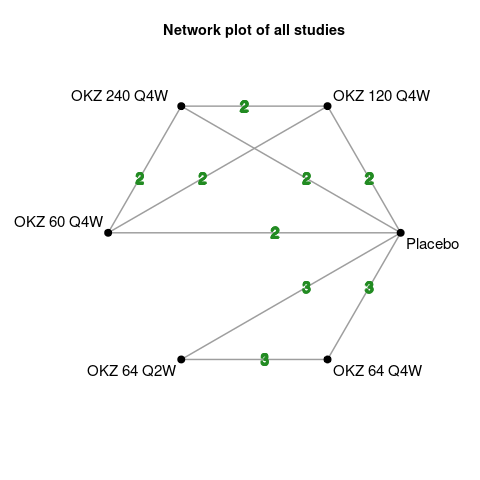


Figure S11: Network plot of ACR50 response after 24 weeks.


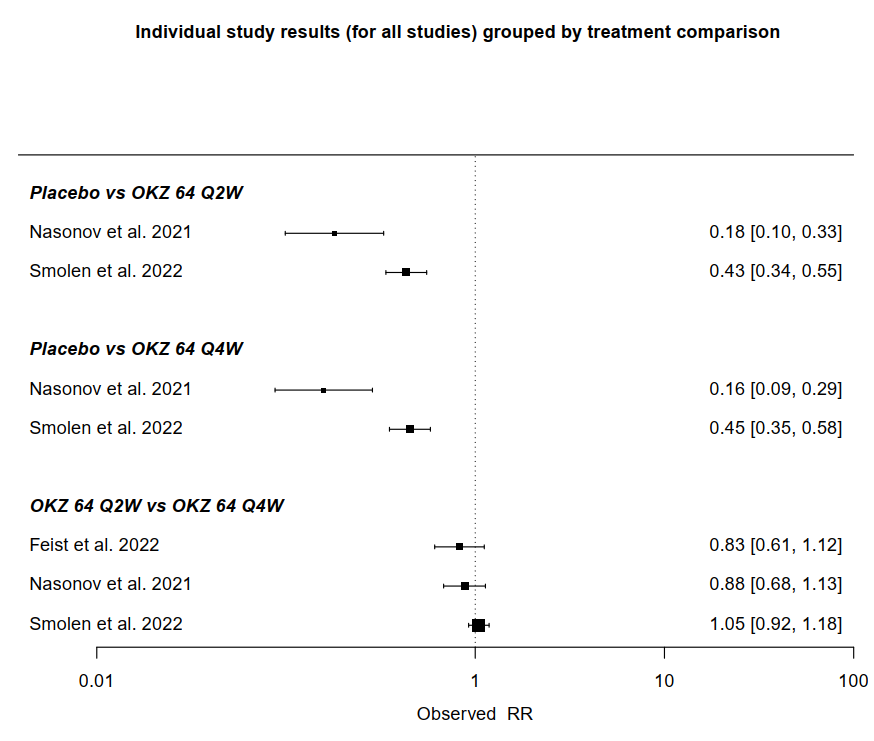


Figure S12: Forest plot of individual study results grouped by treatment component for ACR50 response after 24 weeks.


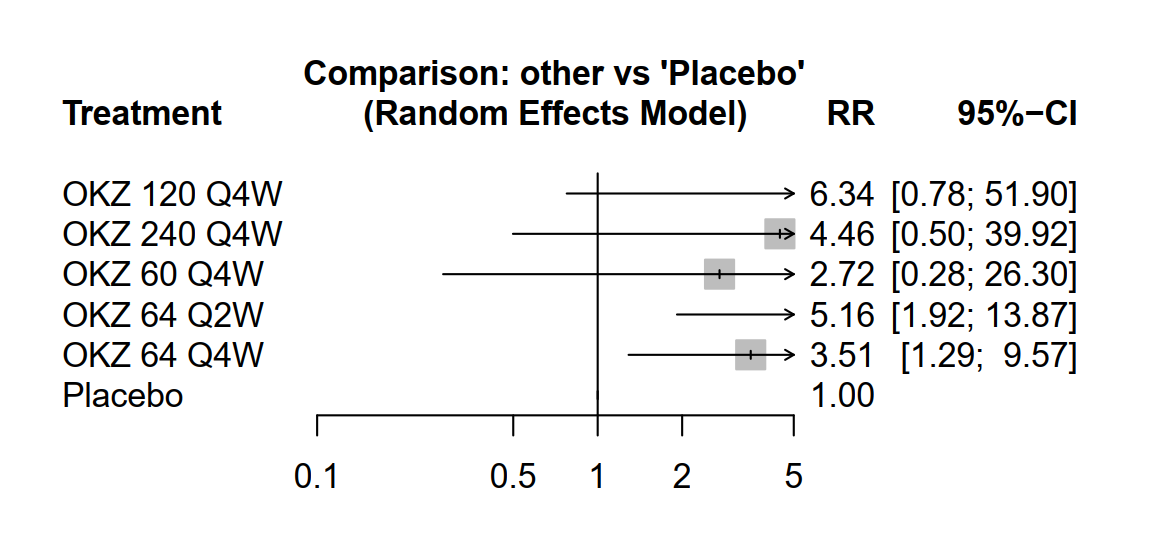


Figure S13: Forest plot of pooled summary estimates derived from network meta-analysis for ACR70 response after 12 weeks.


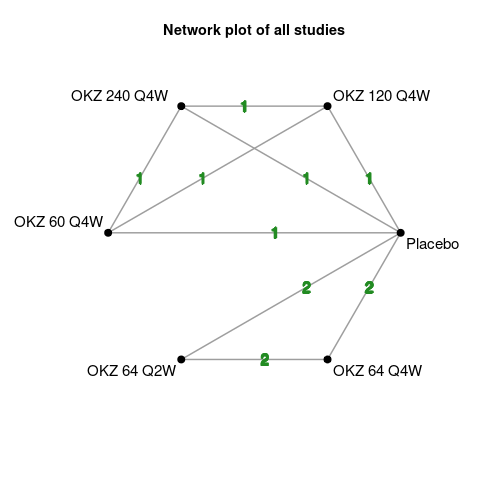


Figure S14: Network plot of ACR70 response after 12 weeks.


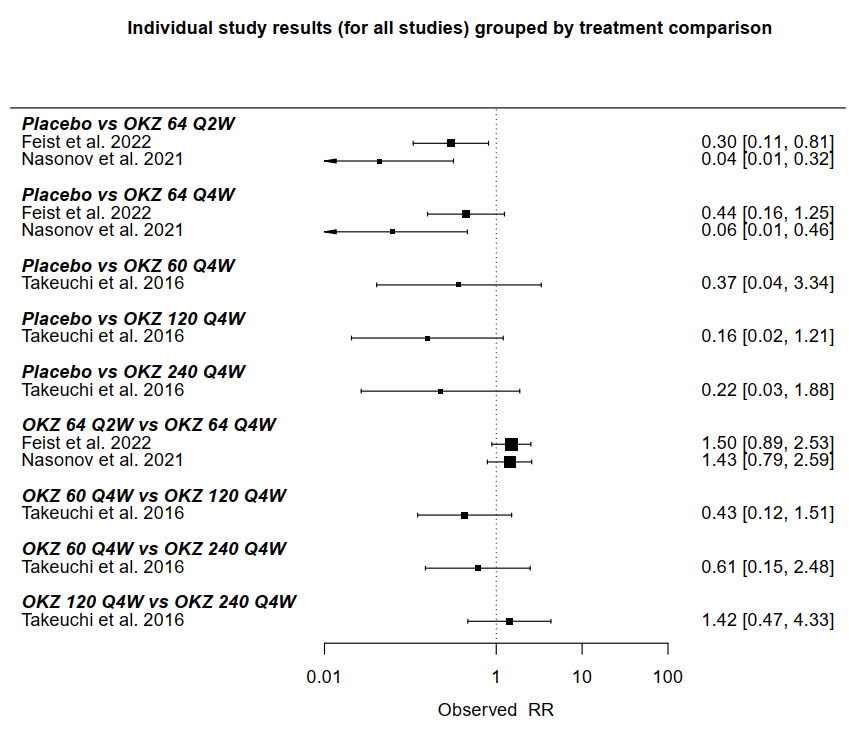


Figure S15: Forest plot of individual study results grouped by treatment component for ACR70 response after 12 weeks.


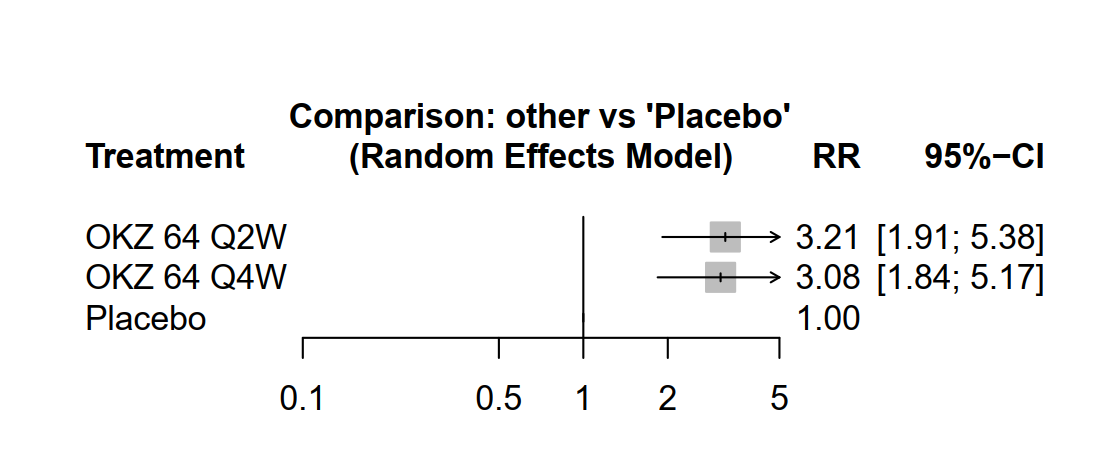


Figure S16: Forest plot of pooled summary estimates derived from network meta-analysis for ACR70 response after 24 weeks.


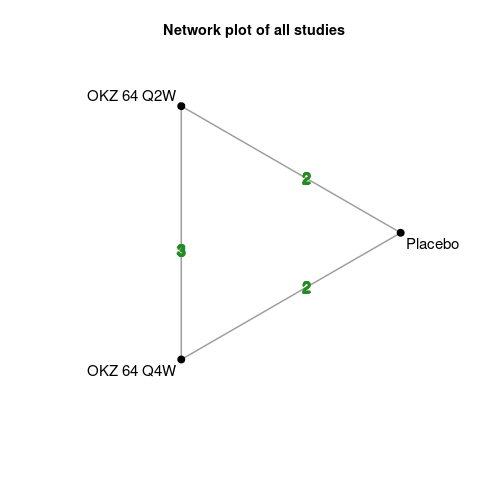


igure S17: Network plot of ACR70 response after 24 weeks.


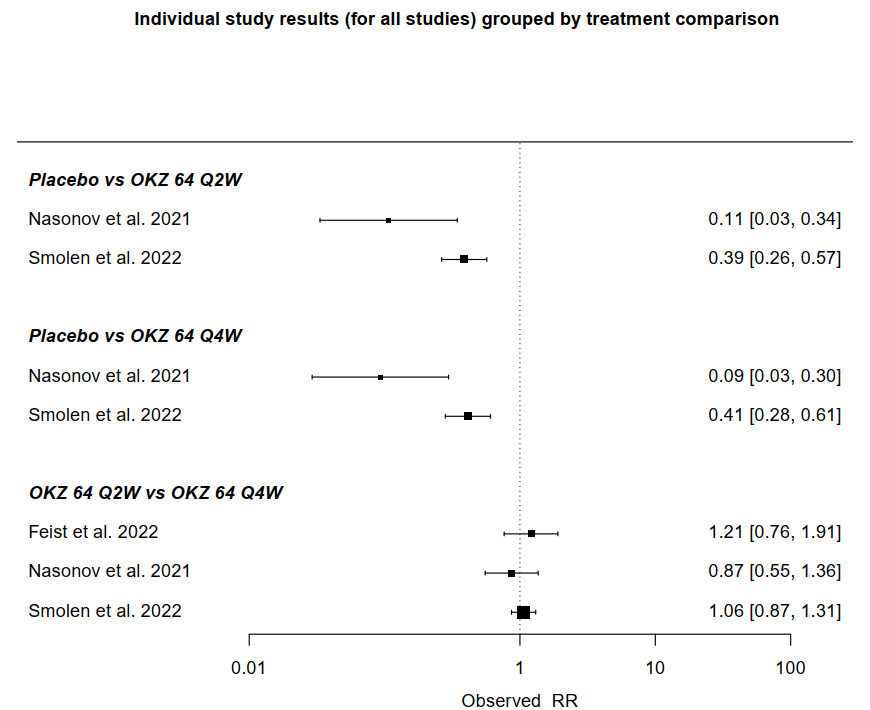


Figure S18: Forest plot of individual study results grouped by treatment component for ACR70 response after 24 weeks.


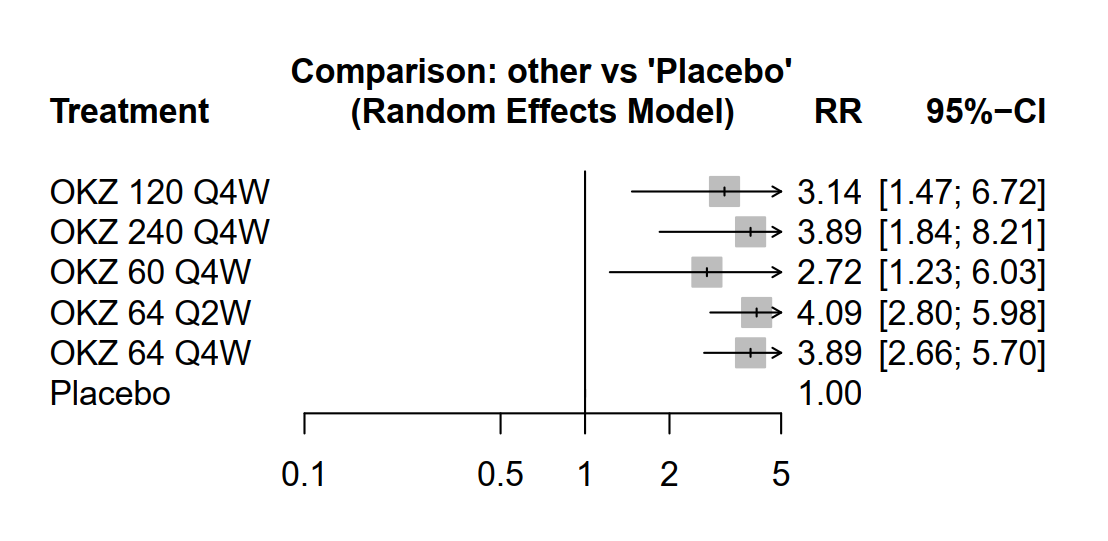


Figure S19: Forest plot of pooled summary estimates derived from network meta-analysis for DAS28-CRP <3.2 after 12 weeks.


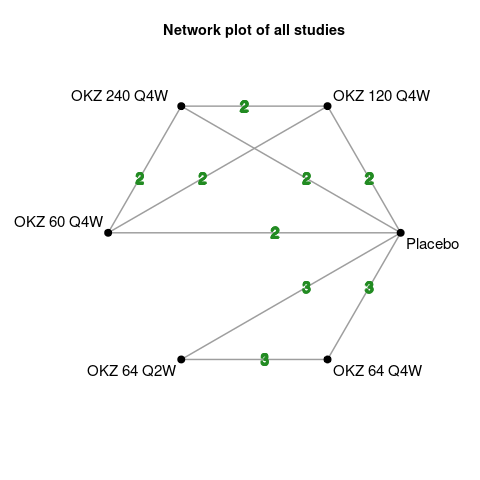


Figure S20: Network plot of DAS28-CRP <3.2 after 12 weeks.


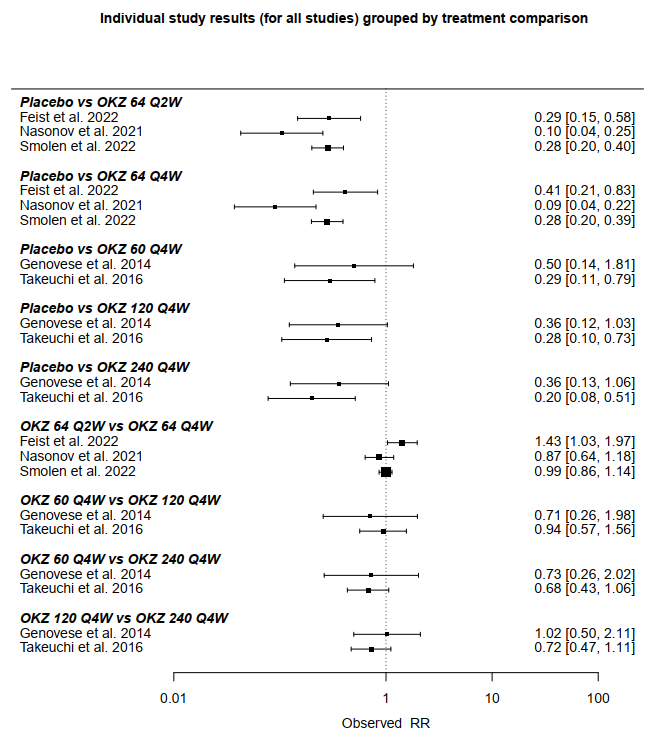


Figure S21: Forest plot of individual study results grouped by treatment component for DAS28-CRP <3.2 after 12 weeks.


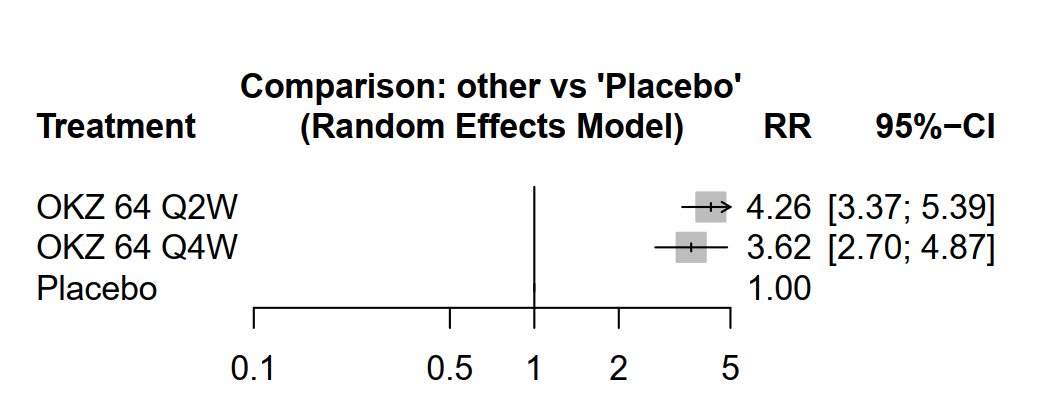


Figure S22: Forest plot of pooled summary estimates derived from network meta-analysis for DAS28-CRP <3.2 after 24 weeks.


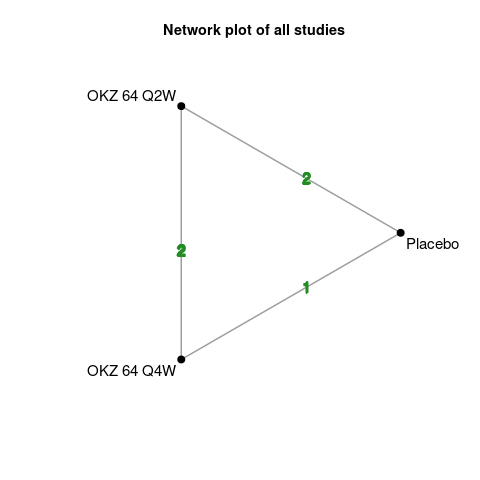


Figure S23: Network plot of DAS28-CRP <3.2 after 24 weeks.


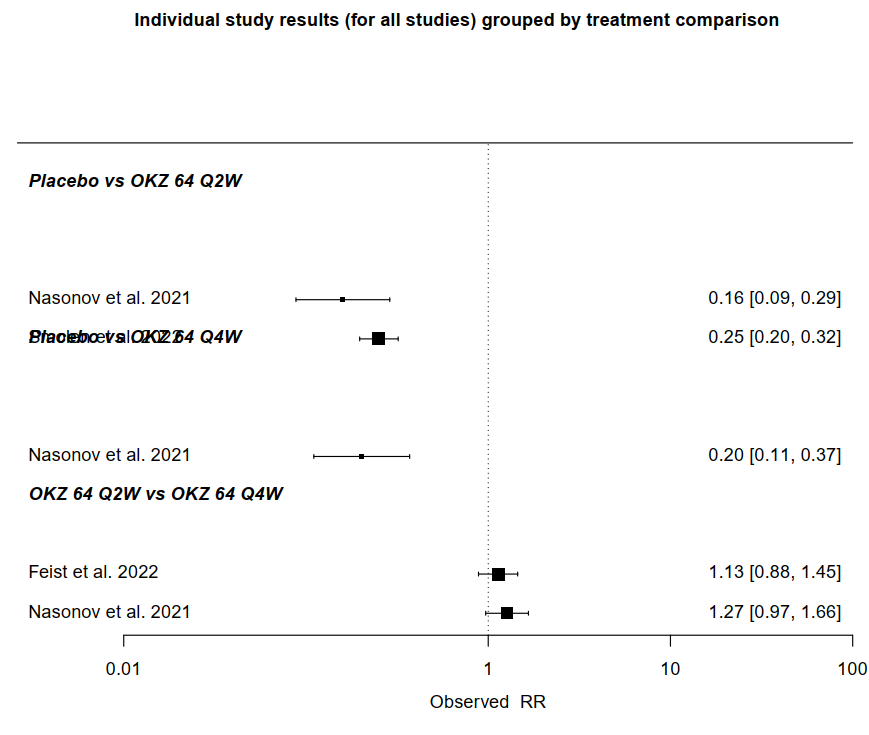


Figure S24: Forest plot of individual study results grouped by treatment component for DAS28-CRP <3.2 after 24 weeks.


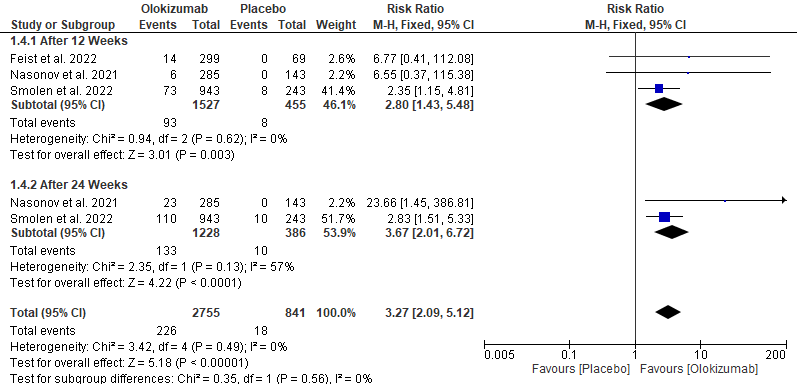


Figure S25: Forest plot of pair-wise analysis of CDAI Score of ≤2.8.


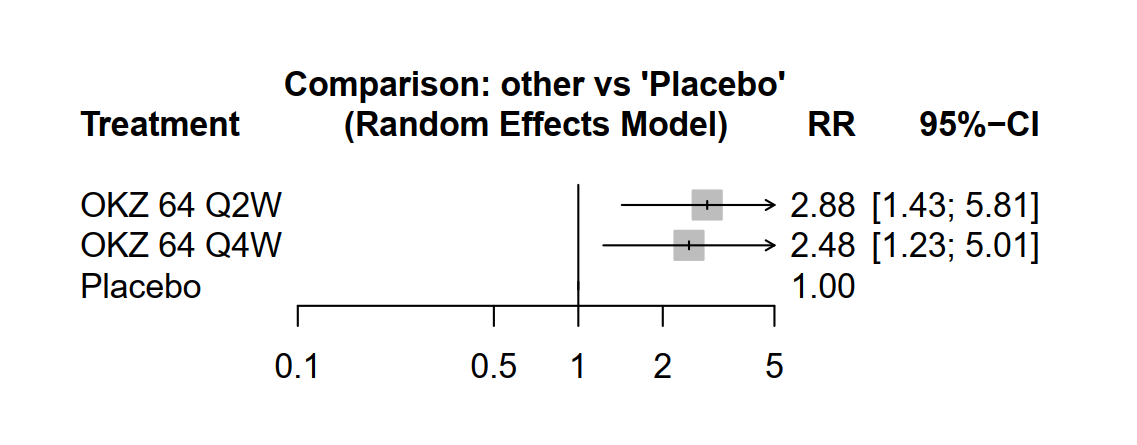


Figure S26: Forest plot of pooled summary estimates derived from network meta-analysis for CDAI Score of ≤2.8 after 12 weeks.


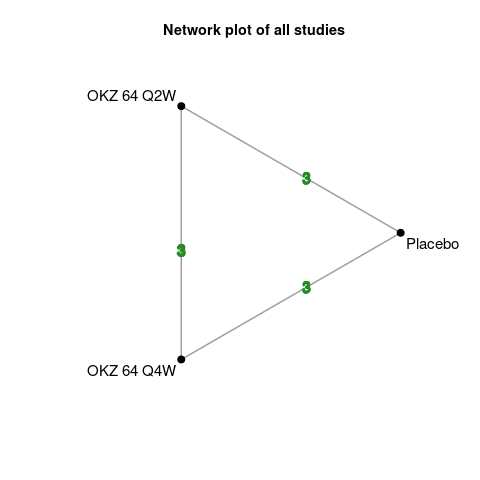


Figure S27: Network plot of CDAI Score of ≤2.8 after 12 weeks.


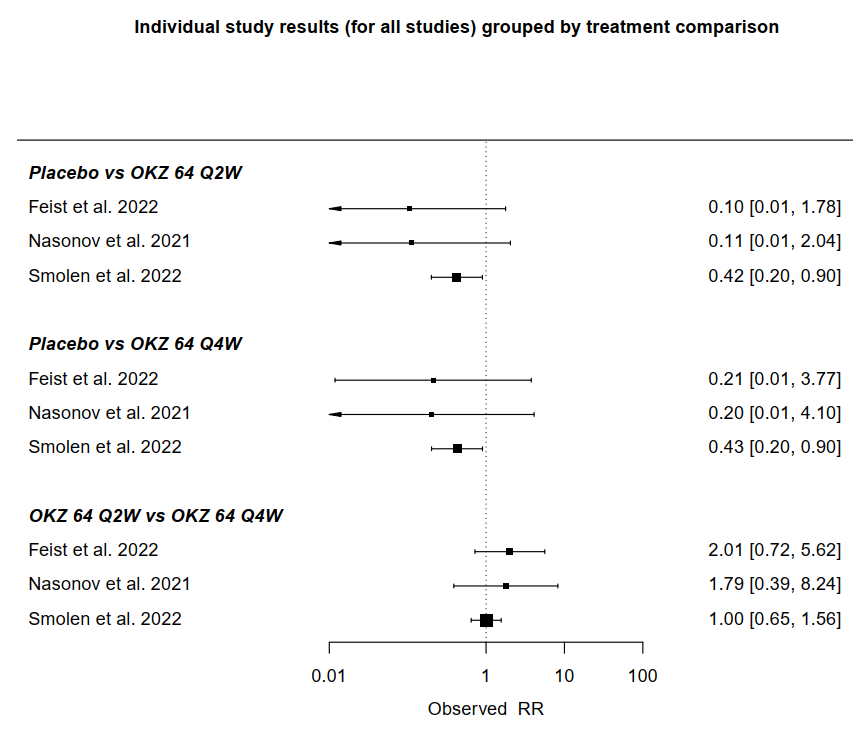


Figure S28: Forest plot of individual study results grouped by treatment component for CDAI Score of ≤2.8 after 12 weeks.


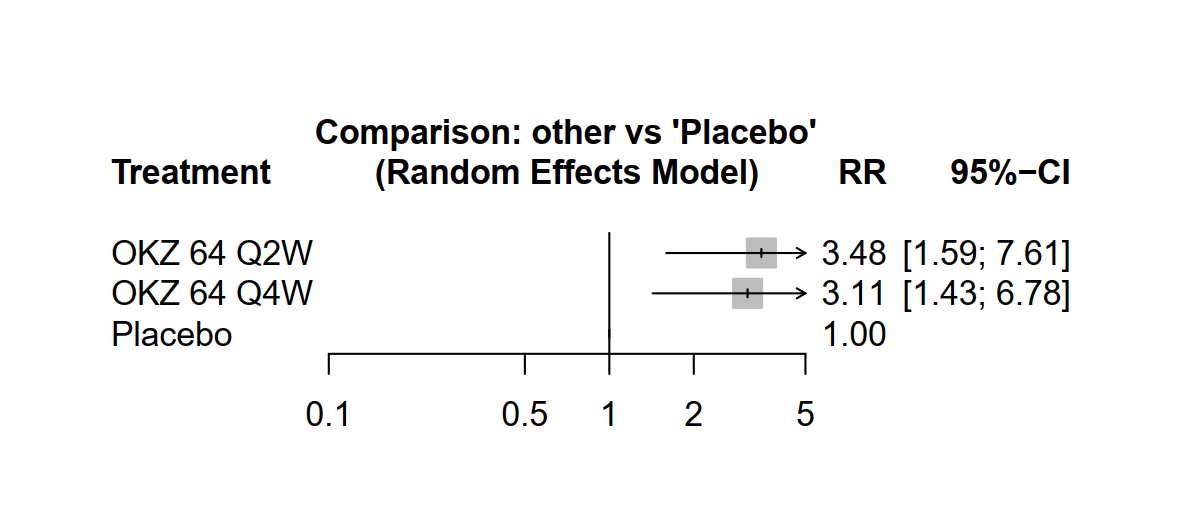


Figure S29: Forest plot of pooled summary estimates derived from network meta-analysis for CDAI Score of ≤2.8 after 24 weeks.


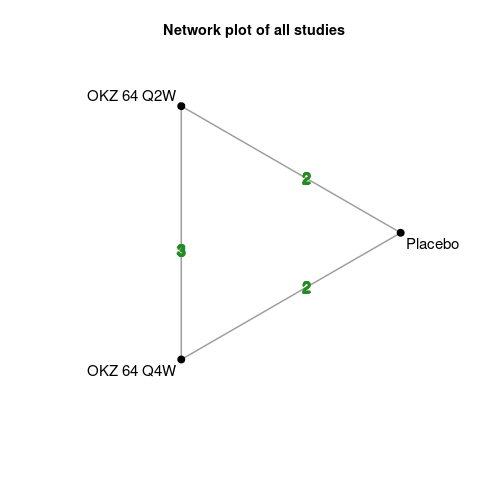


Figure S30: Network plot of CDAI Score of ≤2.8 after 24 weeks.


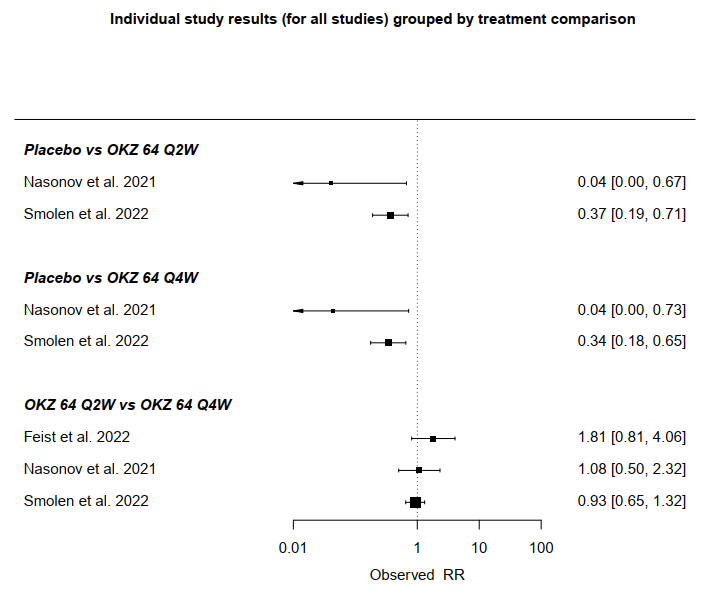


Figure S31: Forest plot of individual study results grouped by treatment component for CDAI Score of ≤2.8 after 24 weeks.


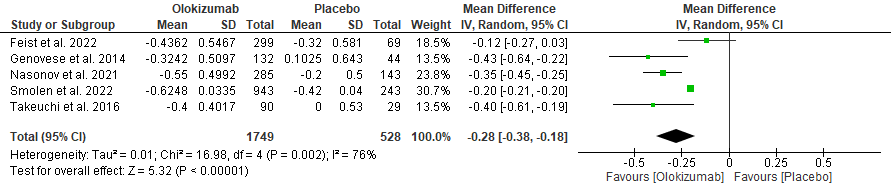


Figure S32: Forest plot of pair-wise analysis of HAQ-DI score change after 12 weeks.


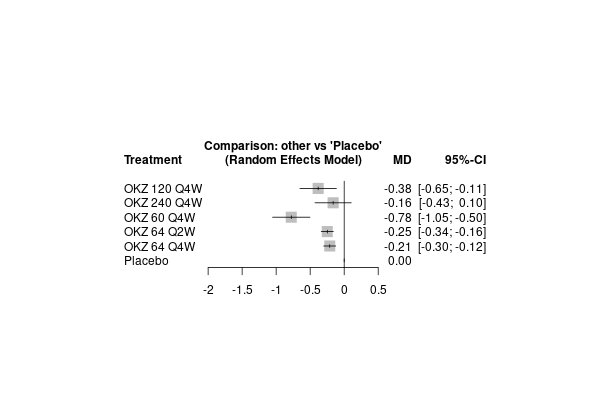


Figure S33: Forest plot of pooled summary estimates derived from network meta-analysis for HAQ-DI score change after 12 weeks.


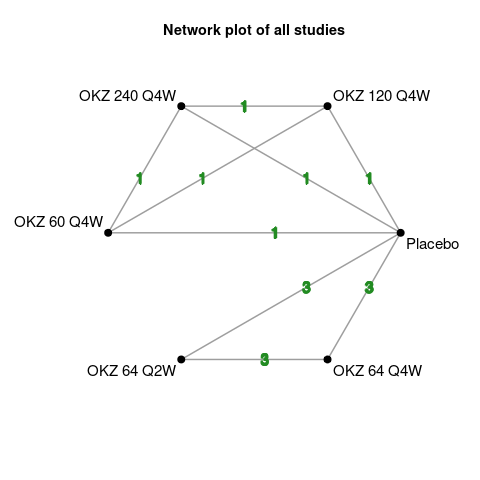


Figure S34: Network plot of HAQ-DI score change after 12 weeks.


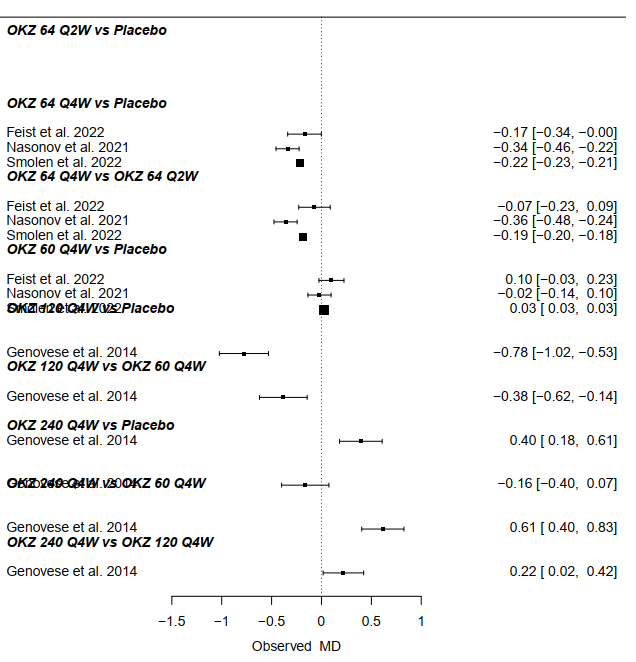


Figure S35: Forest plot of individual study results grouped by treatment component for HAQ-DI score change after 12 weeks.


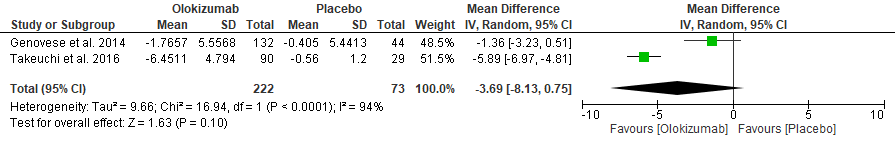


Figure S36: Forest plot of pair-wise analysis of DAS28-ESR score change after 12 weeks.


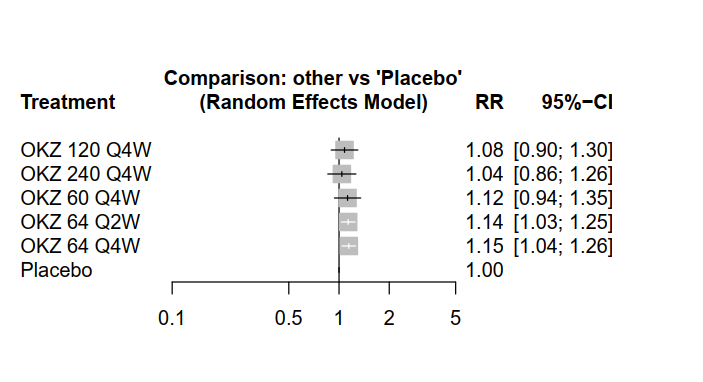


Figure S37: Forest plot of pooled summary estimates derived from network meta-analysis for any TEAEs.


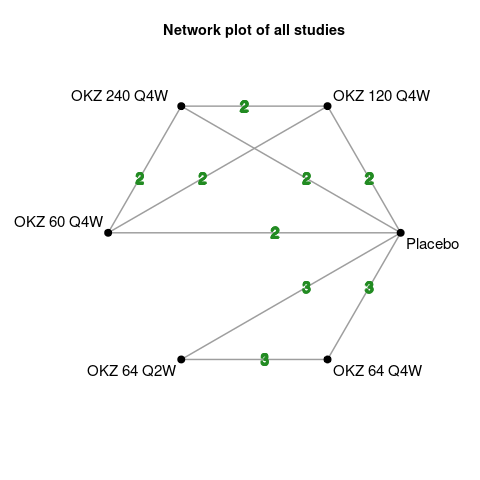


Figure S38: Network plot of any TEAEs.


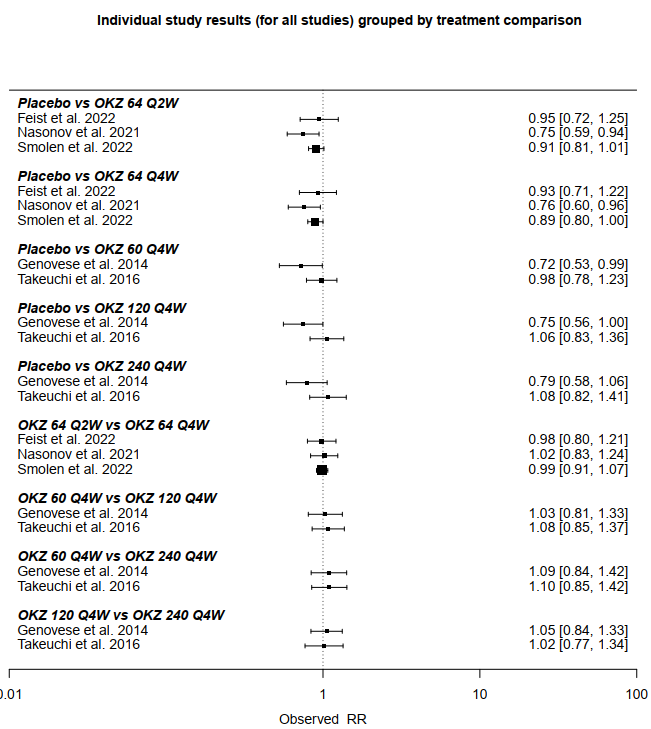


Figure S39: Forest plot of individual study results grouped by treatment component for any TEAEs.


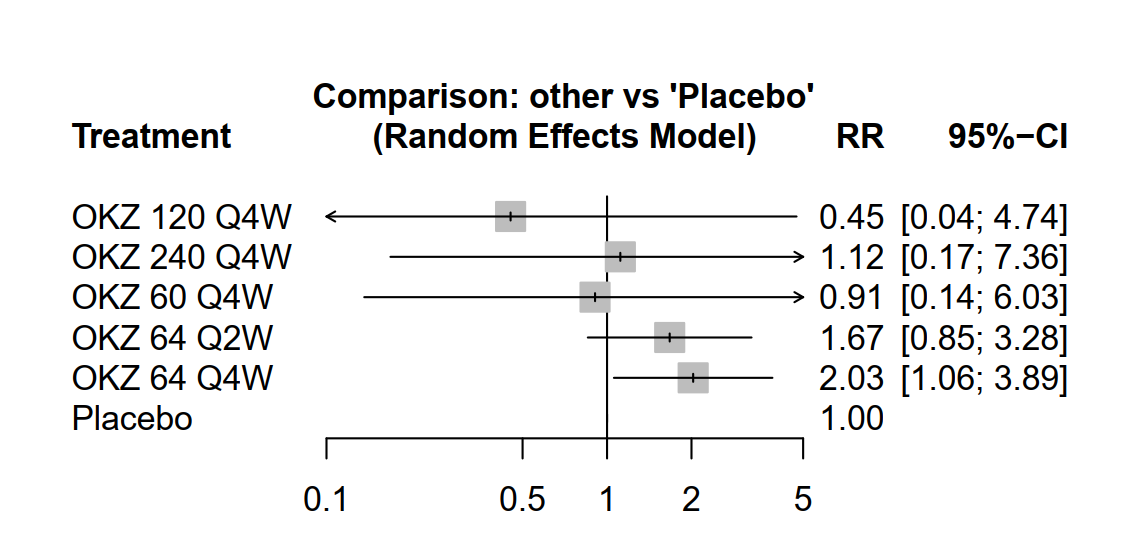


Figure S40: Forest plot of pooled summary estimates derived from network meta-analysis for any TEAEs leading to drug discontinuation.


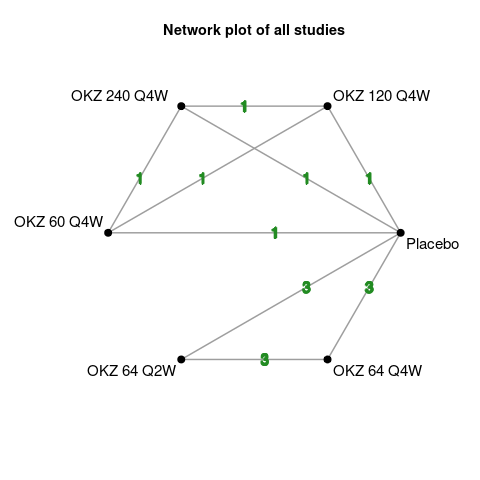


Figure S41: Network plot of any TEAEs leading to drug discontinuation.


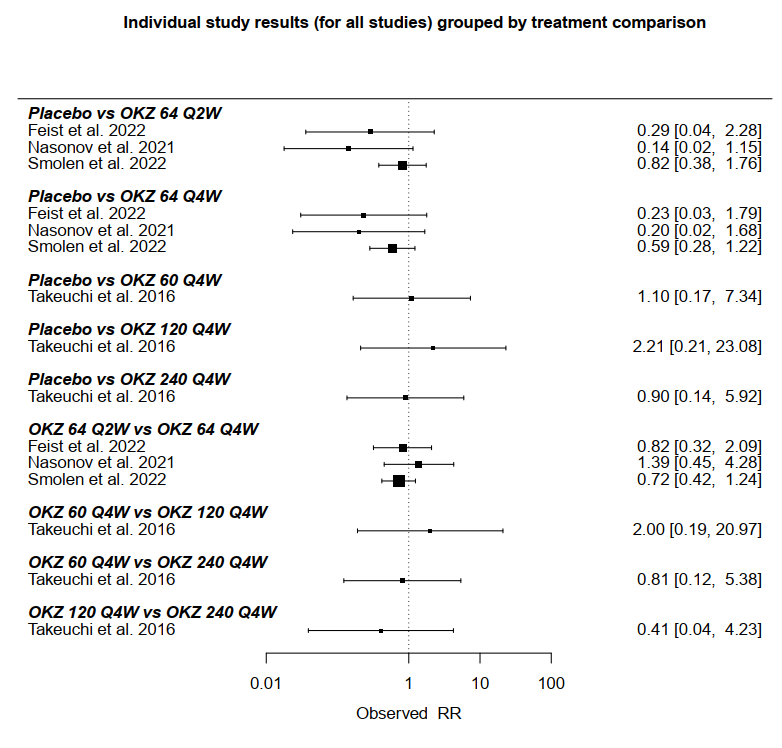


Figure S42: Forest plot of individual study results grouped by treatment component any TEAEs leading to drug discontinuation.


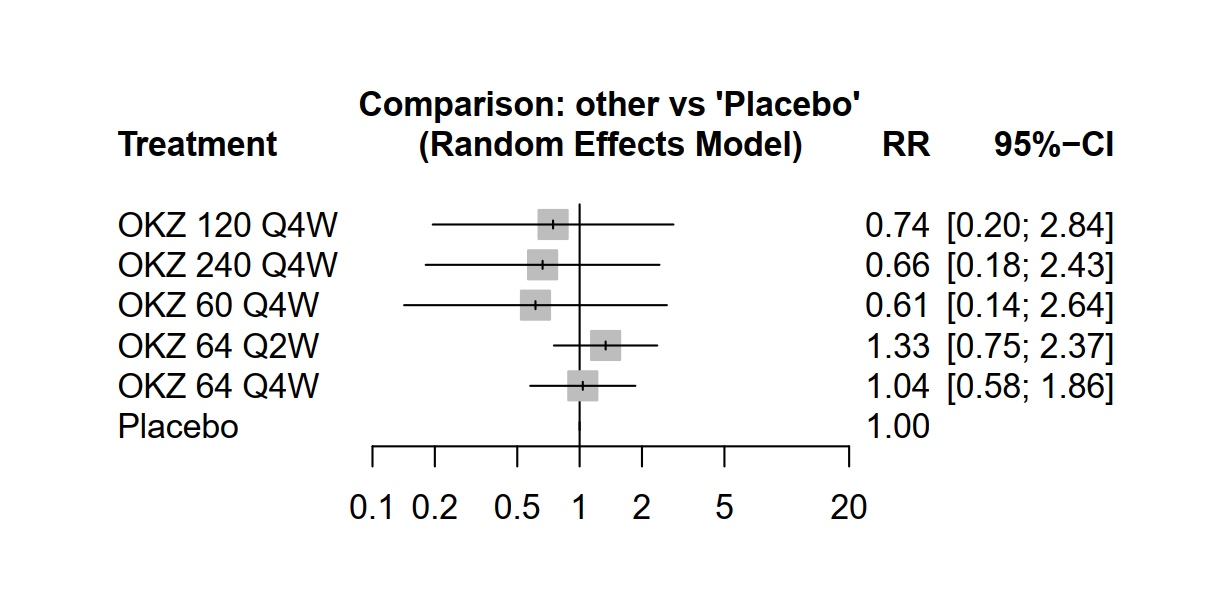


Figure S43: Forest plot of pooled summary estimates derived from network meta-analysis for any TESAEs.


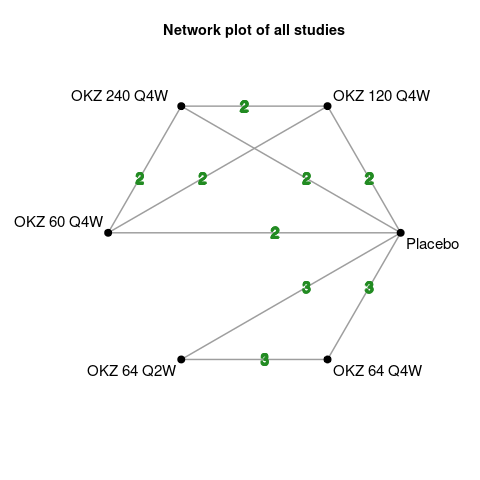


Figure S44: Network plot of any TESAEs.


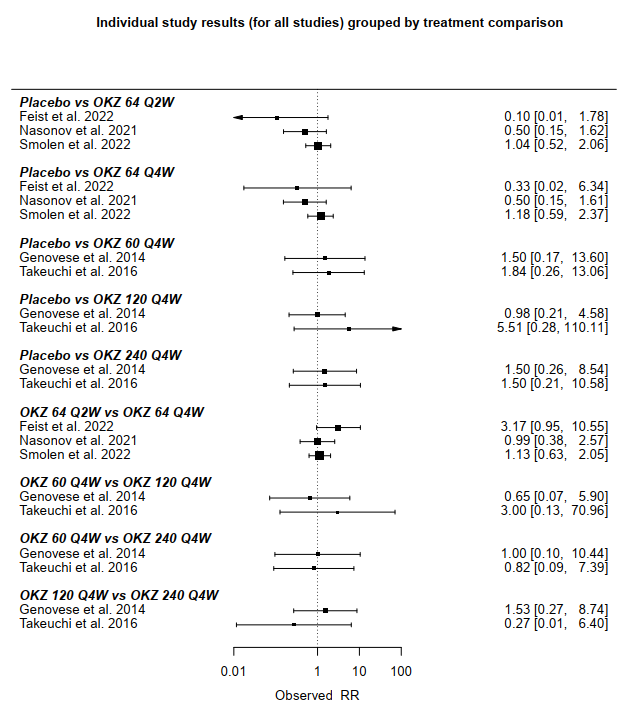


Figure S45: Forest plot of individual study results grouped by treatment component for any TESAEs.


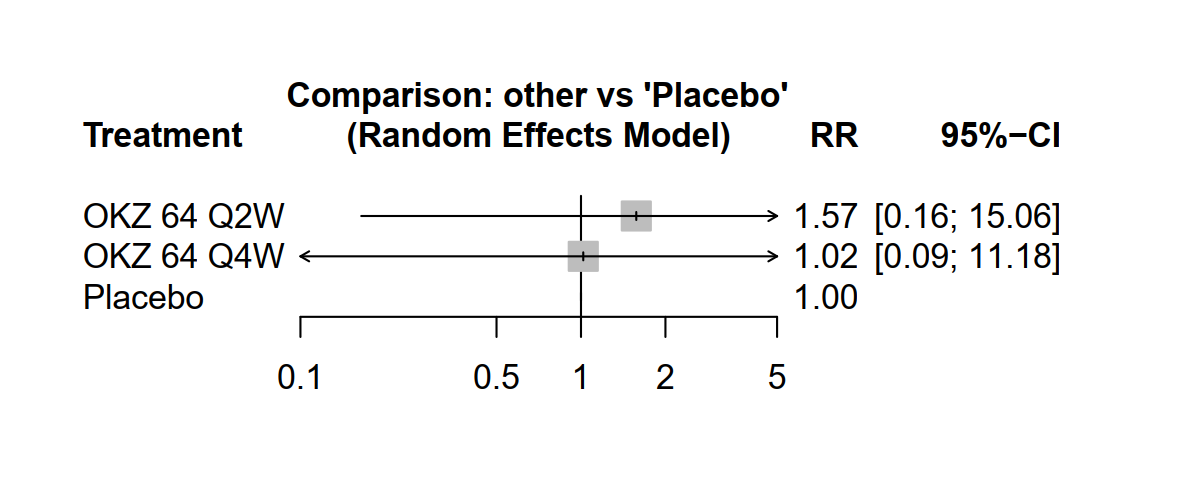


Figure S46: Forest plot of pooled summary estimates derived from network meta-analysis for any-cause mortality.


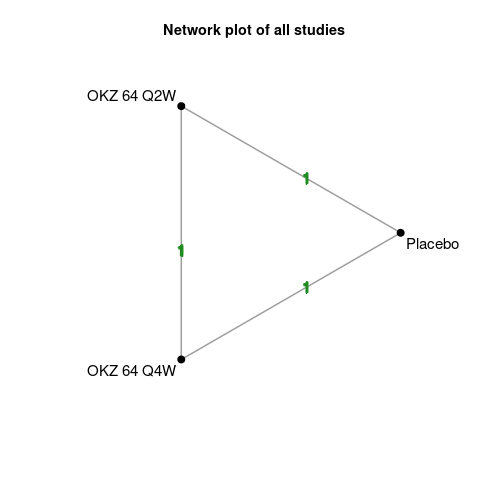


Figure S47: Network plot of any-cause mortality.


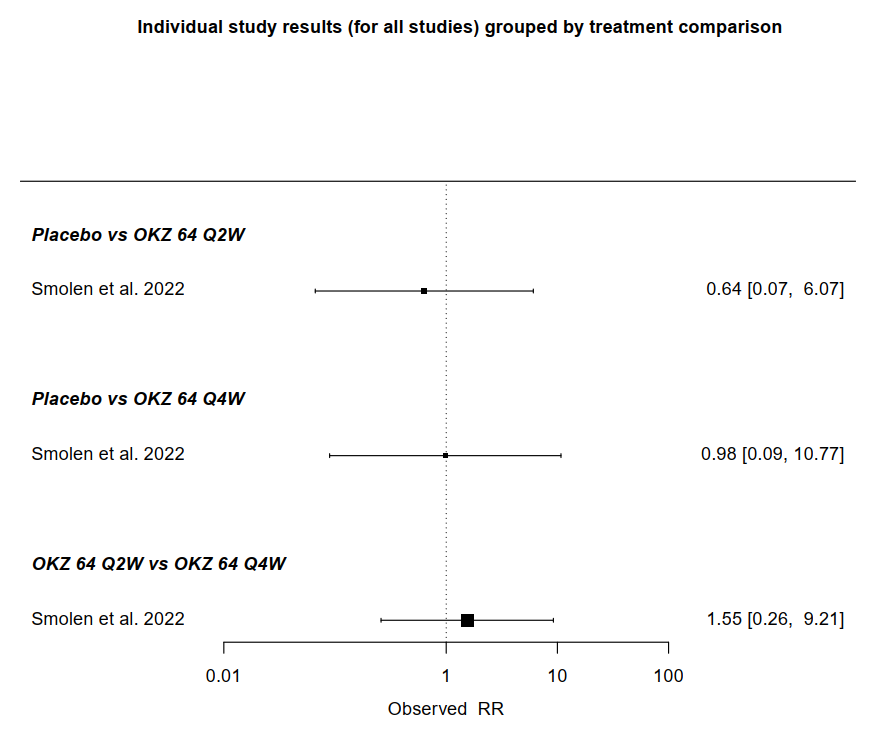


Figure S48: Forest plot of individual study results grouped by treatment component for any-cause mortality


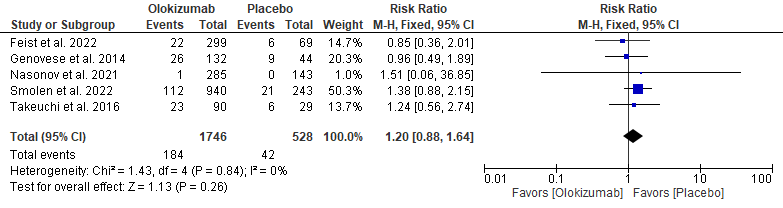


Figure S49: Forest plot of pair-wise analysis of gastrointestinal disorders.


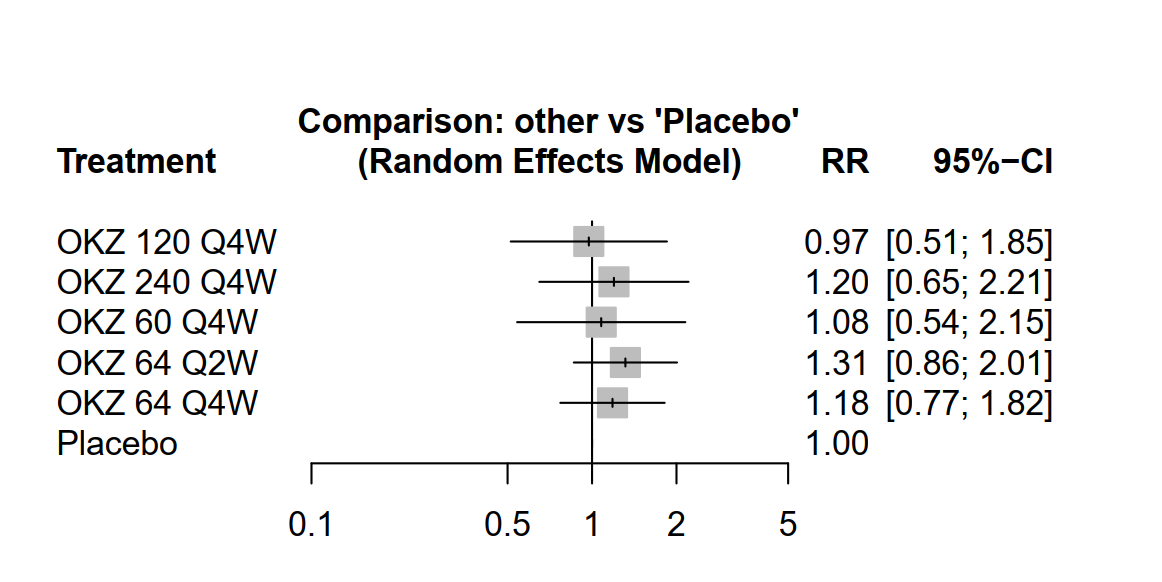


Figure S50: Forest plot of pooled summary estimates derived from network meta-analysis for gastrointestinal disorders.


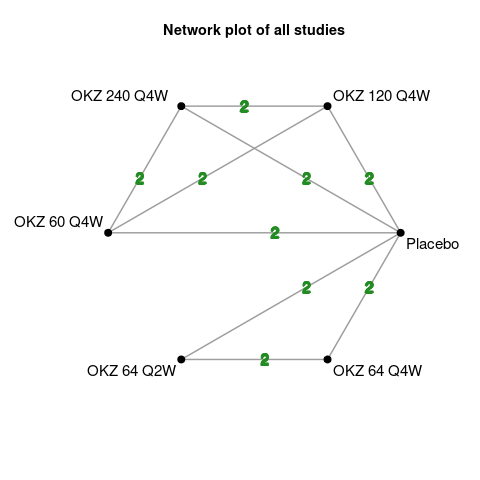


Figure S51: Network plot of gastrointestinal disorders.


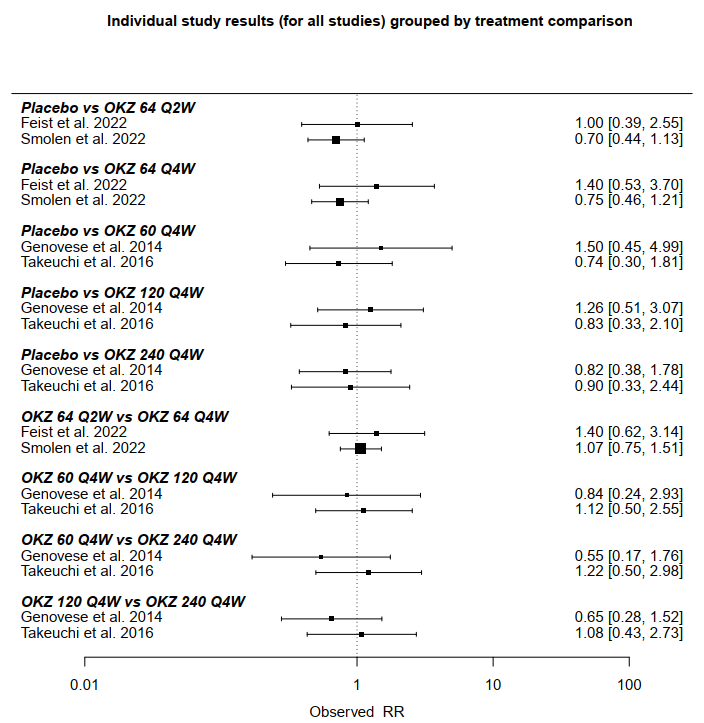


Figure S52: Forest plot of individual study results grouped by treatment component for gastrointestinal disorders.


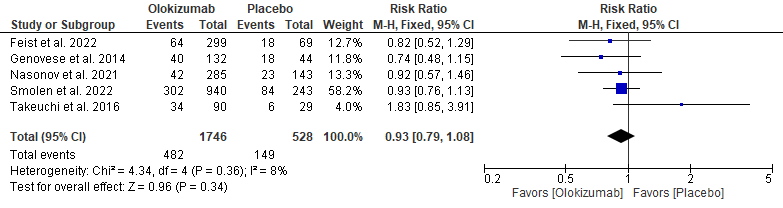


Figure S53: Forest plot of pair-wise analysis of infections.


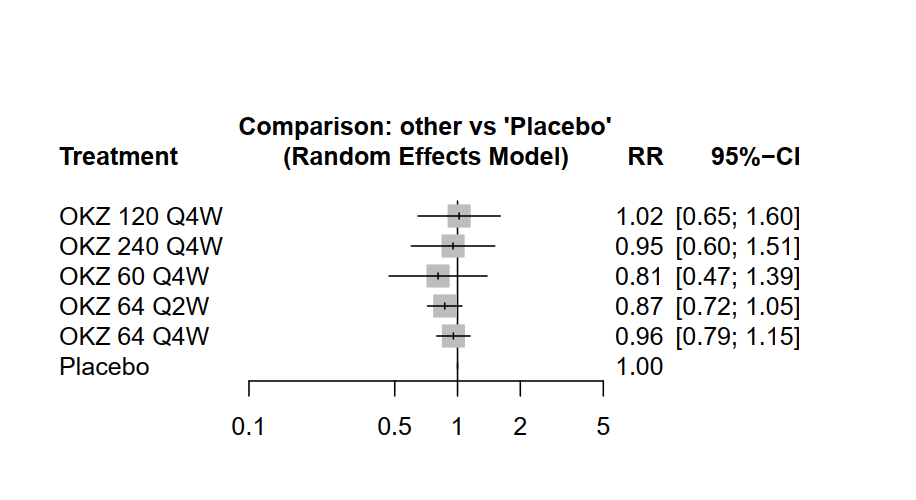


Figure S54: Forest plot of pooled summary estimates derived from network meta-analysis for infections.


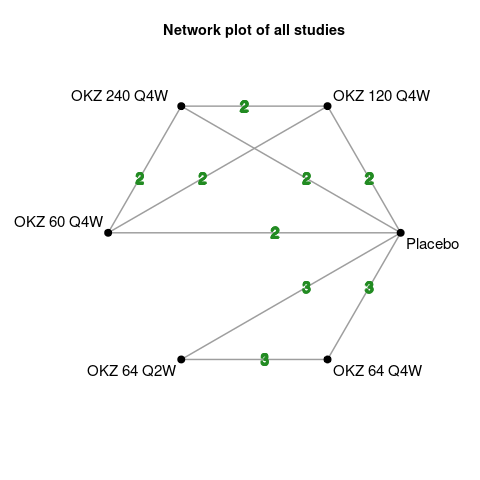


Figure S55: Network plot of infections.


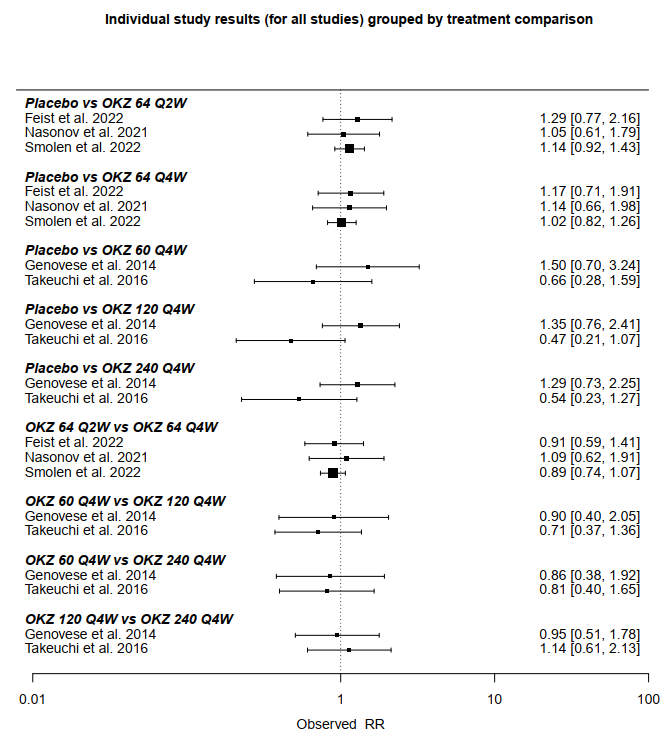


Figure S56: Forest plot of individual study results grouped by treatment component for infections.
